# DNA intermediates of meiotic recombination in synchronous *S. pombe* at optimal temperature

**DOI:** 10.1093/nar/gkt861

**Published:** 2013-10-01

**Authors:** Randy W. Hyppa, Kyle R. Fowler, Lubos Cipak, Juraj Gregan, Gerald R. Smith

**Affiliations:** ^1^Fred Hutchinson Cancer Research Center, Division of Basic Sciences, Seattle, WA, 98109, USA, ^2^Max F. Perutz Laboratories, University of Vienna, Dr. Bohr-Gasse 9, 1030 Vienna, Austria, ^3^Cancer Research Institute, Slovak Academy of Sciences, 83391 Bratislava, Slovak Republic ^4^Department of Genetics, Faculty of Natural Sciences, Comenius University, Mlynska Dolina, 842 15 Bratislava, Slovak Republic

## Abstract

Crossovers formed by recombination between homologous chromosomes are important for proper homolog segregation during meiosis and for generation of genetic diversity. Optimal molecular analysis of DNA intermediates of recombination requires synchronous cultures. We previously described a mutant, *pat1-as2*, of the fission yeast *Schizosaccharomyces pombe* that undergoes synchronous meiosis at 25°C when an ATP analog is added to the culture. Here, we compare recombination intermediates in *pat1-as2* at 25°C with those in the widely used *pat1-114* temperature-sensitive mutant at 34°C, a temperature higher than optimal. DNA double-strand breaks at most hotspots are similarly abundant in the two conditions but, remarkably, a few hotspots are distinctly deficient at 25°C. In both conditions, Holliday junctions at DNA break hotspots form more frequently between sister chromatids than between homologs, but a novel species, perhaps arising from invasion by only one end of broken DNA, is more readily observed at 25°C. Our results confirm the validity of previous assays of recombination intermediates in *S. pombe* and provide new information on the mechanism of meiotic recombination.

## INTRODUCTION

Meiosis, the formation of haploid gametes from diploid precursor cells, involves complex changes to chromosomes and their DNA. During meiosis, recombination between homologous chromosomes (homologs) forms a physical connection that provides tension between homologs on proper attachment to microtubules. This ensures proper segregation of recombined homologs to opposite poles at the first meiotic division. Without recombination and crossovers, in most species, chromosomes often missegregrate, and the resulting aneuploid gametes and subsequent progeny are inviable or debilitated (1). Meiotic recombination also reassorts genetic differences (alleles) between the parental chromosomes, usually giving rise to progeny with genotypes different from the parental genotypes. Recombination thus aids evolution of the species by enhancing genetic variation on which natural selection can act. Understanding the mechanism of recombination is central to understanding this key step in the life cycle of eukaryotes and may lead to ways to prevent birth defects resulting from improper chromosome segregation.

Elucidating the molecular mechanism of meiotic recombination requires obtaining sufficient cells at a given stage of meiosis to allow physical analysis of the DNA intermediates at each stage. Synchronously induced cultures of the fission yeast *Schizosaccharomyces pombe* and the budding yeast *Saccharomyces cerevisiae* have been indispensable in such studies. Particularly useful is the *S. pombe* temperature-sensitive *pat1-114* mutant, which can be synchronously induced by raising the temperature of the culture from 25 to 34°C (2). The higher temperature, however, is near the limit at which meiosis is successful in starvation-induced *pat1^+^* cells, and double-strand break (DSB) formation at 36°C and recombination at 35°C are less frequent than at 25°C (3,4) (unpublished data). Moreover, temperature is an important variable for meiotic chromosomal processes in certain null mutants of *S. cerevisiae* and perhaps wild-type as well (5,6). Thus, results obtained with *pat1-114* cells at 34°C have left uncertain how recombination proceeds at 25°C, the temperature at which many genetic and cytological studies of meiosis have been conducted (7–9).

To overcome this limitation, we created a mutant form of the Pat1 protein kinase (*pat1-as2*) that is inactivated in the presence of an ATP analog, using a procedure successful with many protein kinases (10–13). Wild-type Pat1 is active during vegetative growth of *S. pombe* and indirectly represses the hundreds of genes whose products are required for meiosis (14). When diploid cells heterozygous at the mating-type locus (*h^+^/h^−^*) are starved for nitrogen, Pat1 is inactivated and is unable to phosphorylate and inactivate Mei2, an RNA binding protein that functions in the destruction of meiotic mRNAs during vegetative growth. Nitrogen starvation also activates Ste11, a transcriptional activator of *mei2* and many other meiosis-specific genes (15). Multiple developmental events ensue, culminating in spore (gamete) formation. As *S. pombe* cells starved for nitrogen arrest at the G1 phase of the cell cycle (16), inactivating Pat1 in such cells results in synchronous meiosis: DNA replication occurs between 2 and 3 h in *pat1-114* at 34°C (5–6 h in *pat1-as2* at 25°C), and the first meiotic division (MI) between 5 and 6 h at 34°C (9–10 h at 25°C) (10). DNA can be extracted from such synchronously induced cells to analyze DNA intermediates of recombination.

**Figure 1.**
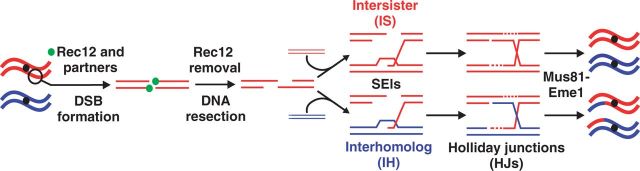
Pathway of meiotic recombination in *S. pombe.* The formation of meiotic DSBs is initiated by the Rec12 protein and several partner proteins. Rec12 breaks the DNA, becomes covalently bound to the 5′ end and is endonucleolytically removed, attached to ∼20 nt of DNA. The 3′ single-stranded tail is lengthened by further 5→3′ nucleolytic resection. One end invades an intact DNA duplex—either the identical sister or the homologous chromatid—and forms an intermediate known as a D-loop or a SEI. Cutting of the displaced strand allows it to anneal with the second initial end at the DSB to form a four-stranded DNA molecule, a Holliday junction (HJ). Resolution of the HJ into two duplex DNA molecules is completely dependent on Mus81-Eme1 and can result in an IH crossover if DSB repair is with the homologous chromosome; repair with the sister chromatid is genetically silent. Each thick line is double-stranded DNA; each thin line is a single strand of DNA.

In *S. pombe* and *S. cerevisiae*, recombination is initiated by the formation of DNA DSBs by Spo11 (called Rec12 in *S. pombe*) (17,18) (Figure 1). As a Spo11 ortholog is encoded by nearly all sexually reproducing species, DSB formation is likely a common or universal feature of meiotic recombination. DSBs do not occur at random across the genome but are much more frequent at certain sites (hotspots) than at others in both yeasts; in *S. pombe*, DSBs occur up to 400 times more frequently at hotspots than in other regions (DSB-cold regions) (19). DSB repair in *S. cerevisiae* occurs by the invasion of one end of the broken DNA into a homologous duplex to form a displacement (D) loop, also called a single-end invasion (SEI); annealing of the other broken end to the displaced strand forms a double Holliday junction (20,21). Cleavage of the displaced strand *before* annealing of the second end would form a single Holliday junction, the most frequent structures observed in *S. pombe* and in a substantial minority of repair events in *S. cerevisiae* (22). Resolution of Holliday junctions (HJs), whether single or double, can produce either a crossover (i.e. with exchange of parental DNA flanking the DSB) or a non-crossover (i.e. with DNA exchanged only near the DSB but not in the flanking regions).

Repair of a DSB with the homolog can produce a genetically recombinant crossover, whereas repair with the sister chromatid cannot. Although it is often stated that meiotic DSB repair occurs with the homolog, e.g. (21,23), HJ-forming repair events with the sister chromatid outnumber those with the homolog by ∼3 or 4:1 at the two DSB hotspots examined in *S. pombe* (22), and recent observations suggest there is more intersister (IS) repair in *S. cerevisiae* than previously thought (24). Indirect evidence suggests that repair of DSBs in DSB-cold regions occurs primarily or exclusively with the homolog in *S. pombe* (25), as appeared to be the case for the DSB hotspots examined in *S. cerevisiae* (26).

The data on *S. pombe* DNA intermediates cited earlier in the text come from studies using *pat1-114* mutants at 34°C. The *pat1-as2* mutant enabled us to test whether meiotic DNA events occur similarly at the more nearly optimal temperature of 25°C. We find that most events are the same, but some distinct differences illuminate aspects of meiotic recombination not previously observed.

## MATERIALS AND METHODS

### *Schizosaccharomyces pombe* strains and genetic methods

The strains used and sources of alleles are listed in Table 1. Construction of the *pat1-as2* strain is described in the Supplementary Figure S4. Standard molecular and genetic procedures and media for growth were used (7,33). Transformation of *S. pombe* with plasmids for deletion and integration was performed using a lithium acetate method (10).

**Table 1.** *Schizosaccharomyces pombe* strains

Strain	Genotype^a^
GP1979	*h^−^/h^−^ ade6-52/ade6-M26 lys3-37/+ +/ura1-171 pro1-1/+ pat1-114/pat1-114 end1-458/end1-458*
GP5082	*h^−^/h^−^ ade6-216/ade6-210 pat1-114/pat1-114 +/ura1-61 mbs1-24/mbs1-25 mus81::kanMX6/mus81::kanMX6 his4-239/+ +/lys4-95*
GP5086	*h^−^/h^−^ ade6-216/ade6-210 pat1-114/pat1-114 +/ura1-61 mbs1-24/mbs1-25*
GP6656	*h^−^/h^−^ ade6-3049/ade6-3049 bub1-234/+ +/vtc4-1104 pat1-114/pat1-114 lys3-37/+ +/ura1-61 mbs1-24/mbs1-25 his4-239/+ +/lys4-95*
GP6657	*h^−^/h^−^ ade6-3049/ade6-3049 bub1-234/+ +/vtc4-1104 pat1-114/pat1-114 lys3-37/+ +/ura1-61 mbs1-24/mbs1-25 mus81::kanMX6/mus81::kanMX6 his4-239/+ +/lys4-95*
GP6232	*h^−^/h^−^ ade6-3049/ade6-3049 pat1-114/pat1-114 rec12-201::6His2FLAG/rec12-201::6His2FLAG his4-239/+ +/lys4-95*
GP7347	*h^−^/h^-^ ade6-3049/ade6-3049 bub1-234/+ +/vtc4-1104 pat1::natMX4/pat1::natMX4 pat1-as2*(L95A)*::hphMX4/pat1-as2*(L95A)*::hphMX4 lys3-37/+ +/ura1-61 mbs1-24/mbs1-25 his4-239/+ +/lys4-95*
GP7348	*h^−^/h^−^ ade6-3049/ade6-3049 pat1::natMX4/pat1::natMX4 pat1-as2*(L95A)*::hphMX4/pat1-as2*(L95A)*::hphMX4 rec12-201::6His2FLAG/rec12-201::6His2FLAG his4-239/+ +/lys4-95*
GP7380	*h^−^/h^−^ ade6-3049/ade6-3049 bub1-234/+ +/vtc4-1104 pat1::natMX4/pat1::natMX4 pat1-as2*(L95A)*::hphMX4 pat1-as2*(L95A)*::hphMX4 lys3-37/+ +/ura1-61 mbs1-24/mbs1-25 his4-239/+ +/lys4-95 mus81::kanMX6/mus81::kanMX6*
GP7381	*h^−^/h^−^ ade6-M26/ade6-52 arg1-14/+ +/lys3-37 +/ura1-171 pat1::natMX4/pat1::natMX4 pat1-as2*(L95A)*::hphMX4/pat1-as2*(L95A)*::hphMX4*
GP7390	*h^+^ ade6-3049 pat1::natMX4 pat1-as2*(L95A)*::hphMX4 rad50S rec12-201::6His2FLAG*
GP7921	*h^−^/h^−^ ade6-M26/ade6-52 arg1-14/+ +/lys3-37 +/ura1-171 pat1::natMX4/pat1::natMX4 pat1-as2*(L95A)*::hphMX4/pat1-as2*(L95A)*::hphMX4 lys1::bleMX6-mat-Pc*

^a^Alleles other than commonly used autoxtrophies are described in the following references: *ade6-3049* (27), *bub1-234* (25), *end1-458* (28), *lys1::bleMX6-mat-P* (10)*, mbs1-24* (29)*, mbs1-25* (29)*, mus81::kanMX6* (30), *pat1-114* (2)*, pat1-as2*(L95A)*::hphMX4* (10)*, pat1::natMX4* (10), *rad50S* (31), *rec12-201::6His2FLAG* (32), *vtc4-1104* (25).

### Analysis of meiotic DSBs and Holliday junctions

Cultures were grown to saturation in EMM2* at 25°C, diluted to OD_600_ = 0.1 in EMM2* and grown overnight at 25°C. When the OD_600_ reached 0.3–0.4, the cells were collected by centrifugation, washed once with water, suspended in EMM2* without NH_4_Cl and incubated at 25°C for 16–18 h. To initiate meiosis, NH_4_Cl was added to 0.5%, and immediately 1-NM-PP1 [4-amino-1-*tert*-butyl-3-(1′-naphthylmethyl)pyrazolo(3,4-d)pyrimidine; Toronto Research Chemicals] was added to 20 μM for *pat1-as2* cultures or the temperature was raised to 34°C without addition of inhibitor for either *pat1-as2* or *pat1-114* cultures. Cells were harvested, DNA extracted and DSBs and HJs assayed (33).

### Analysis of genome-wide DSBs by microarray hybridization

The distribution of DSBs was assayed genome-wide by measuring the positions of Rec12-FLAG covalently self-linked to DNA, i.e. without artificial cross-linking (32). Rec12-FLAG was immunoprecipitated using monoclonal anti-FLAG M2 antibody (Sigma-Aldrich) from extracts of meiotically induced strains; the DNA was amplified, labeled and subjected to microarray hybridization on a 44K *S. pombe* Tiling Array (Agilent). Whole genome data are in Supplementary Figures S1 and S2, and raw data are at http://www.ncbi.nlm.nih.gov/geo/. To allow comparisons between strains in the same experiment, the log_10_ value of each data point (median-normalized IP:WCE ratio) was multiplied by a value ranging from 1.1 to 1.3, to make coincident the levels of six prominent DSB hotspots, shown by Southern blots to be indistinguishable, with the values of the array with the highest values at those sites. Data analyses used R (http://www.r-project.org/) and Bioconductor (http://www.bioconductor.org/).

## RESULTS

### *pat1-as2* meiosis is proficient in DSB formation and repair

**Figure 2.**
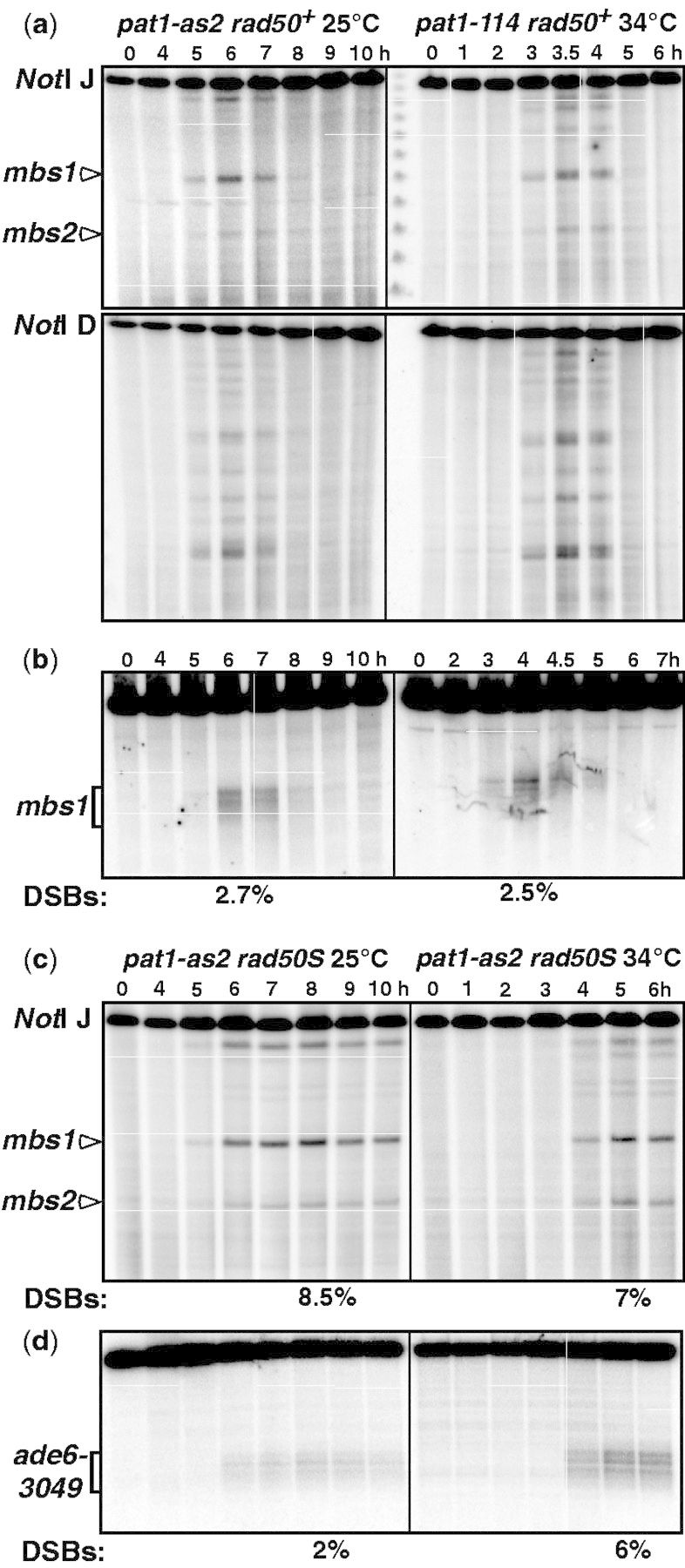
DSB formation and repair at 25°C in *pat1-as2* are comparable with those at 34°C in *pat1-114*. DNA was prepared at the indicated time after meiotic induction of strain GP7348 (*pat1-as2*), GP1979 (*pat1-114*) or GP7390 (*rad50S pat1-as2*), digested with the indicated enzyme, and subjected to gel electrophoresis and Southern blot hybridization with the indicated probe. (**a**) *Not*I-digested DNA probed on the left end of the 0.5 Mb fragment J (upper panels) and the right end of the 1.2 Mb fragment D (lower panels). The middle lane contains concatemers of phage λ DNA (48.5 kb; lowest band is the monomer). (**b**) *Mlu*I-digested DNA probed on the left end of the 24 kb fragment containing *mbs1* (major DSB site on *Not*I fragment J). (**c**) *Not*I fragment J was analyzed as above, using DNA from strain GP7390 (*rad50S pat1-as2*) induced at 25°C with addition of 20 μM 1-NM-PP1 and at 34°C without addition of analog; *pat1-as2* and *pat1-114* are both temperature-sensitive and behave similarly at 34°C (10) (Figure 3 and Supplementary Figures S1–S3). In the examples shown, DSBs appear slightly later in *pat1-as2* at 34°C (c, right) compared with *pat1-114* at 34°C (a and b, right), but this variation of DSBs’ first appearance between 3 and 3.5 h at 34°C has been seen previously (34,35). (**d**) *Afl*II-digested DNA probed at the right end of the 6 kb fragment containing the hotspot *ade6-3049*. Numbers below the panels are the percentage of total DNA in the DNA broken at *mbs1* (b and c) or at *ade6-3049* (d). *Not*I data for *pat1-114* in (a) and (b) were previously published (19).

To compare the frequency and distribution of DSBs at 25 and 34°C, we induced meiosis in *pat1-as2* cells at 25°C by adding 20 μM 1-NM-PP1 and in *pat1-as2* and *pat1-114* cells by raising the temperature to 34°C (without addition of inhibitor), extracted DNA and digested it with *Not*I restriction enzyme (34). [*pat1-as2* is also temperature-sensitive (10).] Two restriction fragments, called J (500 kb) and D (1.2 Mb), were assayed for DSBs by Southern blot hybridization (Figure 2a). At 25°C, DSBs were first observed at 5 h (i.e. after DNA replication was completed), and DSB repair was completed by 8 h (before the first meiotic nuclear division; MI) (10), whereas at 34°C, DSBs appeared at 3 h and were repaired by 5 h as previously reported (19,22). The most prominent DSB hotspot on *Not*I fragment J, *mbs1*, was also analyzed in finer detail and yielded similar results: there is a cluster of distinct DSB sites within *mbs1* in both conditions (Figure 2b). The frequency and distribution of most DSBs were similar in *pat1-as2* at 25°C and in *pat1-114* at 34°C.

To complement these studies, we analyzed DSBs in strains containing the frequently used *rad50S* allele, in which repair is deficient and DSBs accumulate (34). This allowed visualization of even lower-level DSBs at particular sites and would eliminate potentially asynchronous repair that might alter observed DSB levels. The *pat1-as2* allele, which is temperature-sensitive (10), was used at both temperatures but without inhibitor at 34°C. The results agree with the results in *rad50^+^* strains and indicate that DSBs occur at similar locations at both 25 and 34°C (Figure 2c), although some differences were observed, as noted later in the text. For example, DSBs assayed at the hotspot created by the *ade6-3049* single base-pair mutation showed a strong reduction at 25°C compared with 34°C (both in *rad50S* strains) (Figure 2d), although DSBs at *mbs1* were similar at the two temperatures (Figure 2c).

The *pat1-as2* DSBs assayed at 34°C appeared slightly later than *pat1-114* DSBs (Figures 2a and c), but this is within the experimental variation previously seen in *pat1-114* meioses in which DSBs are first seen at 3 or 3.5 h (34,35); DNA replication at 34°C, measured by flow cytometry, was comparable with both *pat1* alleles (10); (unpublished data). DSB formation and repair occur ∼2 h later at 25°C than at 34°C; this delay corresponds to the delay in both DNA replication and the meiotic divisions (10). Addition of the inhibitor to the *pat1-as2* strain at 34°C caused a delay in replication of ∼1 h compared with both *pat1-114* and *pat1-as2* without inhibitor (unpublished data), suggesting the slower meiosis at 25°C likely reflects the effects both of lower temperature and of the ATP-analog inhibitor. As expected, in each case, DSBs were repaired before MI begins, as measured previously (10).

### The genome-wide distributions of meiotic DSBs at 25 and 34°C differ at a few distinct sites

**Figure 3.**
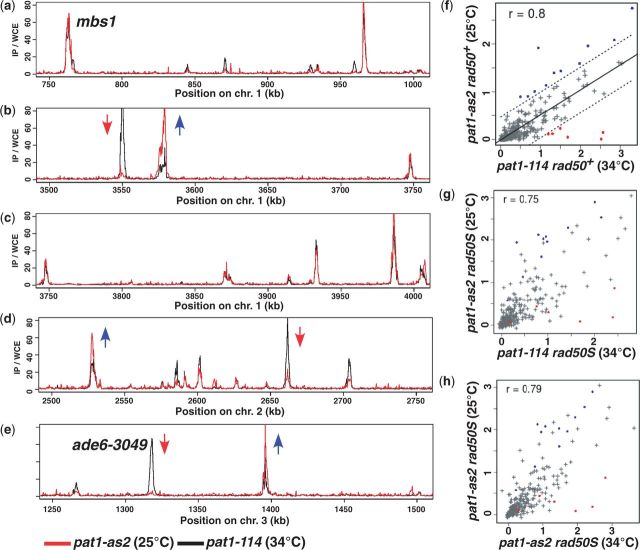
DSB profiles at 25°C in *pat1-as2* and at 34°C in *pat1-114* meioses differ significantly at only a minority of sites. DSBs were assayed as Rec12-DNA complexes by immunoprecipitation (IP), without artificial cross-linking, of Rec12-FLAG from *rad50^+^* strains GP6232 (*pat1-114* at 34°C, black line) and GP7348 (*pat1-as2* at 25°C, red line), amplification of the covalently self-linked DNA and hybridization to a DNA microarray. IP signals were divided by those from the similarly treated whole-cell extract (WCE), median normalized and plotted for the genome regions indicated; data were further normalized by multiplying the log_10_ of the *pat1-as2* signals by 1.1 so that peaks at six DSB sites, shown by Southern blot analysis to be broken to nearly the same extent at the two temperatures (unpublished data), coincided. The genomic regions shown (**a–e**) illustrate both DSB hotspot similarities (e.g. *mbs1* in a, and hotspots in c) and differences (DSB increases indicated by blue arrows, and decreases by red arrows). (**f–h**) Integrated microarray values at 288 DSB hotspots from *pat1-as* meiotic inductions at 25°C were plotted against values from the *pat1-114* (f and g) or *pat1-as2* (h) meiosis done concurrently at 34°C; data are plotted as arbitrary units of Rec12-FLAG DSB signal. Dotted lines are the limits of the 95% prediction interval; *r* is the Pearson correlation coefficient. Blue dots indicate 11 hotspots with significantly increased DSB frequency (outside the 95% prediction interval), and red dots indicate six DSB hotspots with reduced DSB frequency, at 25°C in *pat1-as2* compared with 34°C in *pat1-114* (Figure 3f). Most of the DSB hotspot differences observed in *rad50^+^* strains (f) were also observed *rad50S* strains (g and h). The DSB hotspots with higher DSB levels in *pat1-as2* at 25°C than in *pat1-114* at 34°C (blue dots) not shown in Figure 3a**–**e are on Chr1 at 1150, 1905, 1955, 2520, 3300 and 3985 kb and on Chr2 at 525 and 3010 kb. The DSB hotspots with lower levels (red dots) not shown in Figure 3a**–**e are on Chr2 at 3530 and 3640 kb and on Chr3 at 1835 kb. Data for the entire genome are in Supplementary Figures S1 and S2.

To analyze the meiotic distribution and relative frequency of DSBs across the entire *S. pombe* genome, we immunoprecipitated the genetically active Rec12-FLAG protein covalently self-linked to DNA, which was analyzed by amplification and hybridization to a DNA microarray (ChIP–chip analysis) (32). Chromatin was harvested from *pat1-as2* and *pat1-114* in *rad50^+^* cells at 6.5 h and 3.5 h, and in *rad50S* cells at 5 h and 9 h after induction of meiosis, when Rec12-DNA complexes have maximal abundance (Figure 2a and c) (19). The amount of Rec12-DNA hybridized reflects the frequency of DSB formation; signals at each of the ∼44 000 probes on the microarray were normalized to DNA from the whole-cell extract and further normalized to the genome median value (Figure 3a–e, Supplementary Figures S1 and S2). To aid comparison of results from two or more strains, we multiplied the normalized log_10_ values by an appropriate value (ranging from 1.1 to 1.3) so that the signals at six strong DSB hotspots, as determined by Southern blot hybridizations, were nearly equal (see ‘Materials and Methods’ section). Two independent inductions of the *pat1-as2* strain at 25°C gave highly reproducible results, as did side-by-side inductions of *pat1-as2 rad50S* and *pat1-114 rad50S* strains at 34°C (Supplementary Figure S3a and b; Pearson correlation coefficients *r* = 0.98 and 0.95, respectively).

The data (Figure 3) show that, although most of the distributions and relative frequencies of DSBs are similar in the two conditions, there are some key differences. We considered 288 total hotspots, those with >0.3% DNA breakage as calibrated by direct Southern blot hybridization analyses of 25 DSB hotspots (32). For the vast majority of these 288 hotspots, the amount of breakage was nearly equal in *pat1-as2* at 25°C and *pat1-114* at 34°C in two independent comparisons (*r = *0.80 and 0.81; Figure 3f and Supplementary Figure S3c). Eleven hotspots, however, reproducibly had significantly more DSBs (i.e. outside the 95% prediction interval) in *rad50^+^* strains at 25°C than at 34°C (blue data points in Figure 3f–h and Supplementary Figure S3, some of which are marked with blue arrows in Figure 3b, d and e). Most of these eleven hotspots were also significantly increased in *rad50S* strains (Figure 3g); thus, these sites have increased DSB formation, not slower repair, at 25°C. At this temperature, there was also one new DSB hotspot near 1875 kb from the left end of chromosome 2 (Supplementary Figures S1 and S2).

More interestingly, six intense DSB hotspots at 34°C were reproducibly strongly *reduced* at 25°C (red data points in Figure 3f–h and Supplementary Figure S3), some of which are marked with red arrows in Figure 3b, d and e). One such hotspot is that created by *ade6-3049*, a single bp mutation that creates a binding site for the transcription factor Atf1-Pcr1 (27); DSBs at this hotspot were also reduced as measured by direct Southern blot hybridization (Figure 2d). DSBs at most of these six hotspots were strongly reduced in comparisons between *rad50^+^* strains (Figure 3a–f and Supplementary Figure S1) and between *rad50S* strains (Figure 3g and h and Supplementary Figure S2), in which accumulated DSBs give a more reliable estimate of total DSB frequency (34). Considering the 288 hotspots, the DSB values from *pat1-as2 rad50^+^* and *pat1-as2 rad50S* at 25°C were highly correlated (*r* = 0.88; Supplementary Figure S3d), and the six hotspots were within the range of variation, demonstrating that these reductions are not due to timing differences in DSB repair. Furthermore, comparison of the DSBs in *pat1-as2* and *pat1-114* strains at 34°C also showed high correlation of total hotspots with the six hotspots within the range of variation (*r* = 0.95; Supplementary Figure S3b). Thus, DSB formation, not their repair, differs at 25 and 34°C, and these differences are independent of the *pat1* allele used. DSBs at other sites in *pat1-as2* meiosis were also modestly reduced. These small reductions are within the 95% prediction interval and hence cannot be differentiated from random variation, although they may be meaningful. We discuss later the implications of these observations.

### Holliday junction formation and resolution are similar at 25 and 34°C

**Figure 4.**
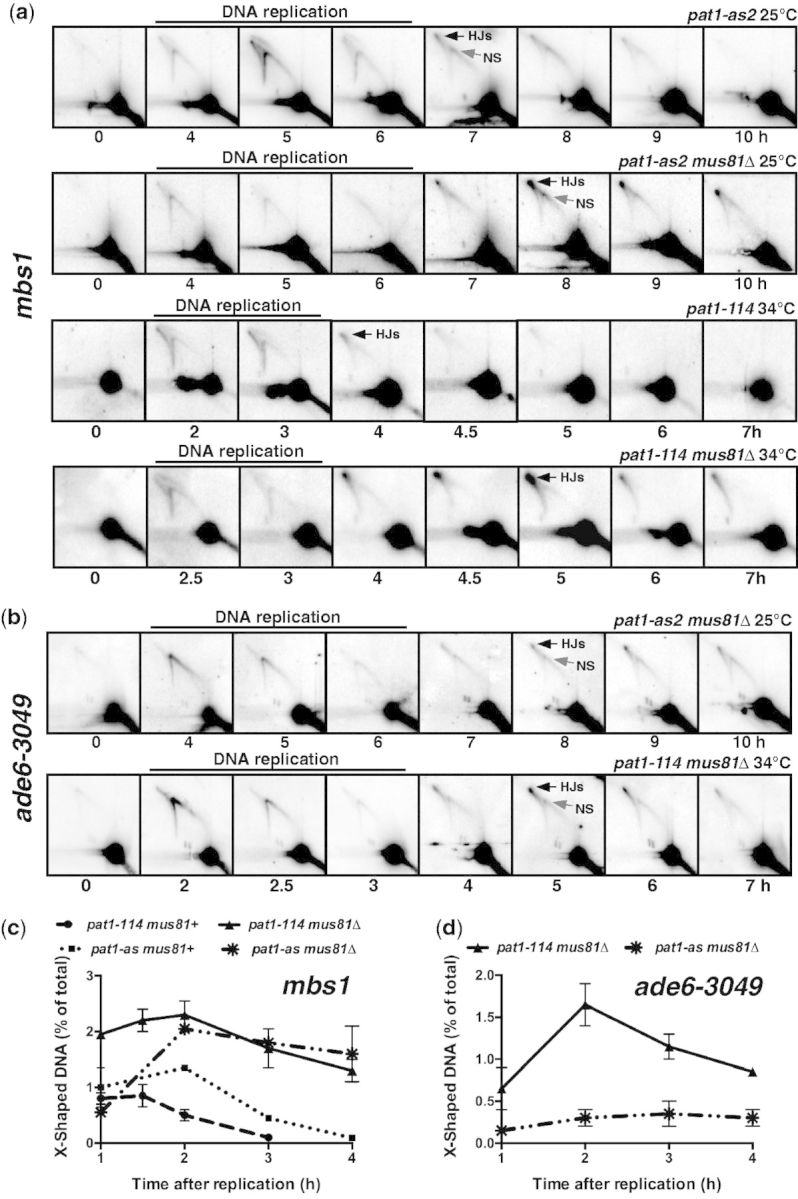
Holliday junction (HJ) formation and resolution at the *mbs1* hotspot at 25°C in *pat1-as2* are comparable with those at 34°C in *pat1-114*, but HJs at the *ade6-3049* hotspot are less abundant. DNA was extracted at the indicated time after induction of meiosis, digested with the indicated restriction enzyme and analyzed by gel electrophoresis and Southern blot hybridization using the indicated probe. The time of DNA replication, as assayed both by DNA content (unpublished flow cytometry data) and by the appearance and disappearance of Y-shaped DNA, is marked on each panel (22). (**a**) HJs assayed on the 10.5 kb *Bsr*GI DNA fragment using a probe at the center of the *mbs1* hotspot. Strains were GP7347, GP7380, GP5086 and GP5082 (top to bottom rows). In *mus81^+^* strains (first and third rows), HJs (black arrow, 7 h top row and 8 h second row) appear at a higher frequency in *pat1-as2* at 25°C than in *pat1-114* at 34°C, likely because of slower kinetics of meiosis at 25°C; accumulated abundance (in *mus81Δ* strains; second and fourth rows) is about the same (see **c**). A novel species (NS; gray arrow, 7 h top row and 8 h second row), perhaps SEI intermediates, in *pat1-as2* was previously obvious in *pat1-114* at 34°C only after psoralen crosslinking (22). (**b**) HJs assayed at the *ade6-3049* hotspot in strains GP7380 (*pat1-as2*; 25°C) and GP6657 (*pat1-114*; 34°C) after digestion with *BsrG*I and using a probe at the center of the *ade6-3049* hotspot (25); the *BsrG*I fragment is 11.8 kb long. Note there are fewer HJs at 25°C (upper row) than at 34°C (lower row). (c) Frequency of HJs observed at *mbs1* in (a) after the completion of DNA replication (3 h in *pat1-as2* and 6 h in *pat1-114*). Error bars indicate the mean ± SEM or range (n = 5 for *pat1-114,* and n = 2 for *pat1-as2*). (**d**) Frequency of HJs observed at *ade6-3049* in (b) after the completion of DNA replication (as in c). Error bars indicate the mean ± range (n = 2 for *pat1-114* and *pat1-as2*). Data for *pat1-114* at *mbs1* were previously published (22).

To investigate further the proficiency of meiosis induced in *pat1-as2* cells, we assayed the formation and resolution of HJs (Figure 1) at the *mbs1* DSB hotspot. As previously observed at 34°C in *pat1-114* cells (22,25), HJs arose after DSB formation and were resolved at about the time that MI began in the *pat1-as2* meiosis [Figure 4a; (10)]. In both cases, HJ resolution depended on the Mus81-Eme1 HJ resolvase; in a *mus81* null background, HJs accumulate, allowing a more accurate measure of the total frequency of HJs. There was no significant difference in the frequency of accumulated HJs (i.e. in *mus81Δ* strains) at the *mbs1* hotspot in *pat1-as2* cells at 25°C and that in *pat1-114* cells at 34°C (2.1 versus 2.3%, Figure 4a and c). At the *ade6-3049* DSB hotspot in *mus81Δ* mutants, there were 4-fold fewer HJs (0.4%) at 25°C in *pat1-as2* meiosis than at 34°C in *pat1-114* meiosis (1.7%, Figure 4b and d). This finding is consistent with the reduction of DSBs observed at the *ade6-3049* hotspot at 25°C compared with 34°C (Figures 2d and 3e).

**Figure 5.**
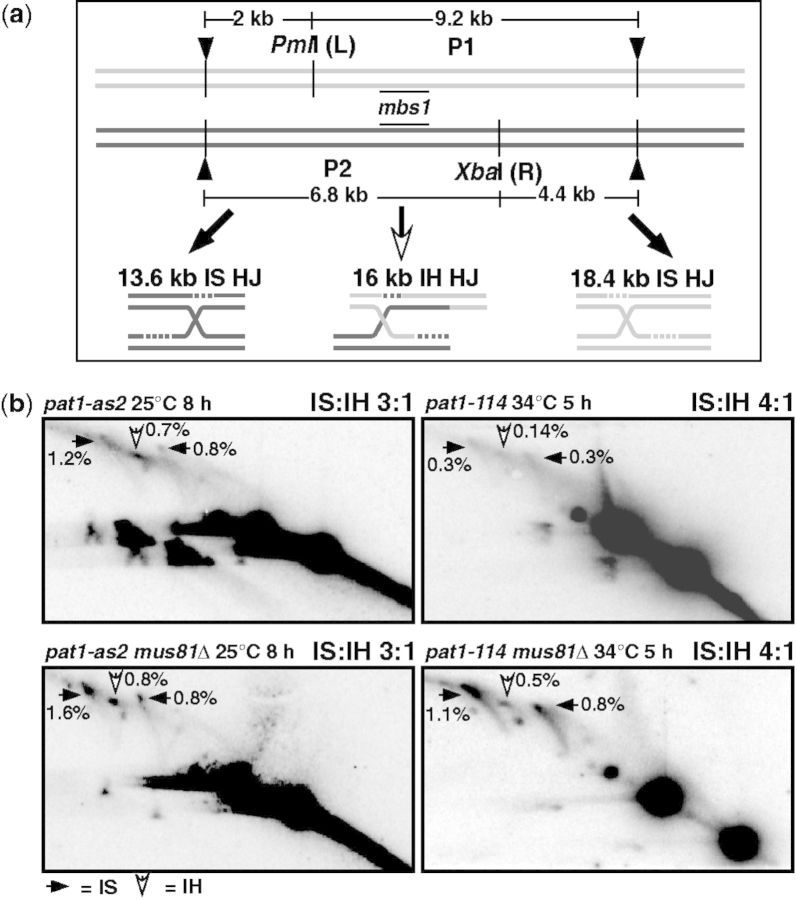
The ratio of IS and IH HJs at the *mbs1* hotspot at 25°C in *pat1-as2* is comparable with that at 34°C in *pat1-114.* (**a**) IS and IH HJs at *mbs1* were assayed by 2D gel electrophoresis of DNA that contained heterozygous restriction site mutations flanking *mbs1* (designated L and R) and digested with *Pvu*II (black arrowheads), *Pml*I and *Xba*I (22) and hybridization using a probe for the central region of *mbs1.* (**b**) Strains used were as in Figure 4a. In both *pat1-as2* and *pat1-114* strains, there are markedly more IS HJs (IS, black arrows) than IH HJs (IH, white arrows), although the IS:IH ratio is slightly lower in *pat1-as2* than in *pat1-114*. Data for *pat1-114* at *mbs1* are from (22).

Chromosome segregation has higher fidelity at 25°C than at 34°C (10). At 34°C IS, HJs outnumber interhomolog (IH) HJs (Figure 1), which could result in low crossover frequency and chromosome missegregation. If so, the IS:IH ratio would be expected to be lower at 25°C. Heterozygous restriction site polymorphisms flanking the *mbs1* hotspot allowed us to measure the relative frequency of IS and IH HJs among the total HJs seen (Figure 5a) (22). The observed ratio of IS to IH HJs at *mbs1* at 25°C in *pat1-as2* was 3:1, consistent with earlier observations of 4:1 at 34°C in *pat1-114* (Figure 5b) (22,25). Similar results were observed at *ade6-3049*: although total HJs were less frequent at 25°C than at 34°C (Figure 4d), the IS:IH ratio was 3:1, as observed before at 34°C (25) (unpublished data).

One advantage of *pat1-as2* meiosis is that it proceeds slower and DNA intermediates of recombination persist longer, allowing a more sensitive measure of the low-level HJs formed in a *mus81^+^* strain. For example, 1.4% of the total DNA was observed as HJs at *mbs1* in *pat1-as2* at 25°C but 0.9% in *pat1-114* at 34°C (Figure 4a and c). This is especially important when assaying IS and IH HJs; both were more frequent in *pat1-as2* at 25°C meiosis than in *pat1-114* at 34°C, although the ratio was similar, as noted earlier in the text (Figure 5b, top panels). The *pat1-as2* meiosis at 25°C also facilitated detection of a novel species running at a position on the 2D gel indicative of Y-shaped DNA, which may be SEI intermediates (Figure 1) (20). This species was not readily seen in *pat1-114* meiosis at 34°C but could be seen in *pat1-as2* meiosis at 25°C in both *mus81^+^* and *mus81Δ* strains (Figure 4a, arrows in 7 h and 8 h panels). A faint signal corresponding to this species in *pat1-114* meiosis at 34°C was more abundant after crosslinking of the DNA with psoralen than without crosslinking (22,25). Shorter duration or lower stability of this species at 34°C may account for its prominence at 25°C without crosslinking. As expected if these DNA species are precursors to HJs, the corresponding species disappeared sooner than HJs: the HJ:species ratio increased from 1.3 to 1.7 to 2.6:1 at 7, 8 and 9 h in *pat1-as2 mus81^+^*. In the *pat1-as2 mus81Δ* strain, the ratio increased from 1.8 to 3.4:1 at 7 and 10 h (Figure 4a). These results provide evidence for a DNA recombination intermediate not previously seen in any organism other than *S. cerevisiae.*

### Genetic recombinant frequencies are mildly increased in *pat1-as2* on addition of an ectopic *mat-Pc* cassette

In a previous study, we showed that spores from meiosis at 25°C had a higher viability and higher frequency of proper chromosome segregation compared with spores from meiosis at 34°C (10). Additionally, ectopic insertion of the *mat-Pc* cassette in a homozygous *h^−^* diploid restored spore viability and chromosome segregation to wild-type (*pat1^+^*) levels. We also observed that *pat1-as2* diploid strains, either at 25 or 34°C, had slightly reduced meiotic recombination (10) (unpublished data). In an attempt to restore full levels of meiotic recombination, the *mat-Pc* cassette was inserted into an *h^−^/h^−^ pat1-as2/pat1-as2* diploid. Addition of the *mat-Pc* cassette increased recombination by a factor of ∼3 at *ade6-M26 x 52* (intragenic recombination; i.e. gene conversion) and by ∼50% between *ade6* and *arg1* (intergenic recombination; i.e. crossing over) (Table 2). Neither increase, however, was statistically significant: for *ade6* intragenic recombination *P* = 0.063 by unpaired *t*-test, and for *ade6 – arg1* intergenic recombination *P* = 0.17 by contingency chi-square test. The higher variance in these data than in those previously published may reflect the occasional meiotic and mitotic instability of the *mat-Pc* cassette, which is flanked by direct repeats (38).

**Table 2.** The *mat-Pc* cassette moderately increases recombinant frequency in *pat1-as2* meiosis at 25°C

*mat1-Pc*^a^	Recombinant frequency	
	*ade6M26* × *ade6-52* (Ade^+^/10^6^ viable spores; mean ± SEM)^b^	*ade6 – arg1* (cM)^c^
−	1200 ± 110 (n = 3)	16
+	3650 ± 1100 (n = 6)	24

^a^The *mat1-Pc* cassette was present (+; strain GP7921) or not (–; strain GP7381).

^b^Recombinant frequencies were based on 51 or more colonies counted. In heterothallic *pat1^+^* crosses, the Ade^+^ recombinant frequency ranges from 2500 to 6400 per 10^6^ viable spores (10,17,25,36).

^c^Recombinant frequencies, based on 58 or more recombinants, were converted to cM using Haldane’s equation. In heterothallic *pat1^+^* crosses, the *ade6 – arg1* genetic distance ranges from 68 cM (37) to 73 cM (36).

### Crossover DNA at the *mbs1* hotspot, but not at *ade6-3049*, is formed equally at 25 and 34°C

**Figure 6.**
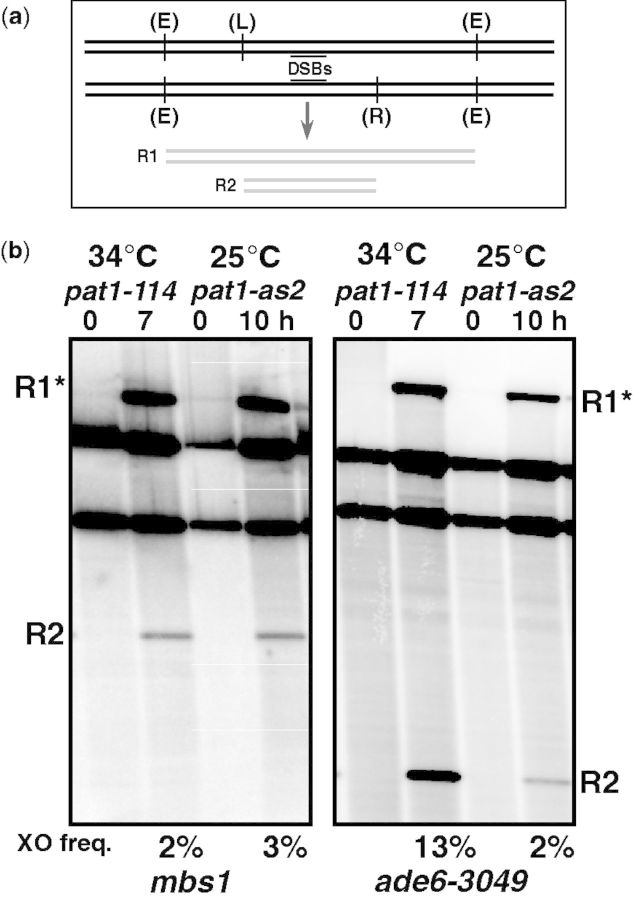
Crossover DNA is equally abundant at the *mbs1* hotspot, but less abundant at the *ade6-3049* hotspot, at 25°C in *pat1-as2* compared with 34°C in *pat1-114.* (**a**) Diagram of the DNA constructs used to measure crossover DNA at *mbs1* and *ade6-3049* using DNA restriction site polymorphisms flanking the hotspots (see Figure 5 for *mbs1* construct). The restriction enzymes used at *mbs1* are *Pvu*II (E), *Pml*I (L) and *Xba*I (R). The restriction enzymes used at *ade6-3049* are *Sca*I (E and L) and *Pml*I (R). (**b**) DNA from strains GP6656 (*pat1-114*) and GP7347 (*pat1-as2*) was extracted at the indicated time after induction of meiosis and analyzed by gel electrophoresis and Southern blot hybridization. Analysis of DNA used probes at the centers of the indicated hotpot (that in Figure 4a for *mbs1* and that in Figure 4b for *ade6-3049*). Crossover frequency, the mean of two experiments, is 2 R2/total DNA, as partial digestion can contribute to R1. R2 is 4.8 kb at *mbs1* and 3.8 kb at *ade6-3049* (22,25).

Using a physical assay, we determined the frequency of recombination between two heterozygous restriction sites closely flanking the *mbs1* hotspot or the *ade6-3049* hotspot. This allowed us to measure the total amount of crossover DNA, rather than just the frequency of recombinants among viable spores (Figure 6a) (22,25). Crossover DNA was measured as twice the frequency of the shorter recombinant restriction fragment R1 (Figure 6a and b) because the larger, though not the smaller, recombinant-length restriction fragment can also arise by incomplete digestion of the DNA. At *mbs1*, the frequency of crossover DNA was 3% at 25°C and 2% at 34°C (Figure 6b), in accord with the similar frequencies of DSBs and HJs at *mbs1* at the two temperatures (Figures 2b and 4c). As expected from the lower frequency of DSBs and HJs at *ade6-3049* at 25°C than at 34°C (Figures 2d and 4d), crossover DNA was also much lower at 25°C than at 34°C, 2 versus 13% in this side-by-side comparison (5.5% in a previous assay of *pat1-114* at 34°C (25). Thus, these data provide a consistent picture of DNA recombination intermediates under the two conditions used here.

## DISCUSSION

The *pat1-as2* mutant studied here provides for the first time a convenient way to prepare large cultures of meiotic *S. pombe* cells synchronously induced at 25°C, a temperature at which many studies of meiosis have been done. Synchronous cultures are essential for biochemical analyses of transient intermediates, and it is important to have similar conditions for both genetic and biochemical assays. The *pat1-as2* mutant affords experimental flexibility and allows comparisons to be made at lower temperature than the *pat1-114* temperature-sensitive mutant previously available. Using this allele, we have found that many features of DNA intermediates of recombination are the same as those in the *pat1-114* mutant at 34°C, the condition previously used to study DNA recombination intermediates. These results confirm many of the conclusions previously drawn, e.g. (22,25,32). Some features of two intermediates—DSBs and joint DNA molecules at certain hotspots—are, however, different at the two temperatures. This difference has helped illuminate the basis of the distribution of DSBs across the genome, a major problem in the field of meiosis, and has pointed to a new intermediate in *S. pombe* meiotic recombination, as discussed later in the text.

At six hotspots, we found that DSBs are strongly reduced (i.e. outside the 95% prediction interval; Figures 3f–g) at 25°C compared with 34°C (Figures 2 and 3). Two of these hotspots, *ade6-3049* and one designated in a previous study 2-2 (39)*,* require the stress-response transcription factor Atf1-Pcr1 for high-level DSB formation (39). Additionally, we found modestly reduced (<2-fold) DSB frequencies at 10 of 15 chromosomal sites with the closest match to the consensus sequence for Atf1-Pcr1 binding (27). Previous DSB analysis of these 15 sites showed a Pcr1-dependent reduction at nine of these sites (39); we found a modest reduction at seven of these nine sites, including *cds1*, at 25°C. However, analysis of the other four strongly reduced DSB hotspots showed no connection to Atf1-Pcr1 activation. These reductions may be partially explained by the presence of genes strongly induced by heat shock that flank five of the six reduced DSB hotspots (40), but some other factor or factors must also affect this reduction, as there are many loci with genes induced by heat shock across the genome that showed no change at lower temperature. Further studies to decipher what these hotspots have in common could lead to important advances in understanding the factors that influence the distribution DSB hotspots.

As expected, the abundance of joint DNA molecules, including HJs, parallels that of DSBs, at least at the two hotspots examined. Comparing abundance at 34°C with that at 25°C, both DSBs and HJs at *mbs1* are approximately equal, but at *ade6-3049* both are reduced at 25°C (Figures 2–4). The reduction at *ade6-3049* at 25°C can be accounted for by its dependence on activated Atf1-Pcr1, as suggested earlier in the text. As the majority of DSB hotspots have indistinguishable levels of DSBs at the two temperatures, we infer that the abundance of HJs is also the same at most hotspots at the two temperatures and that previous conclusions about HJs are valid across the genome, with minor exceptions. The frequency of crossovers generated at the two DSB hotspots also parallels the frequency of DSBs at the two temperatures. At *mbs1*, where DSBs were similar at both 25 and 34°C (Figure 2b and c), crossover DNA measured by physical assay was also similar at the two temperatures (Figure 6b). At *ade6-3049*, however, there was a comparable reduction of both crossover DNA and DSBs at 25°C compared with 34°C (Figures 2d and 6b). These results agree with previous studies of Atf-Pcr1 mutants that showed decreased DSB-formation and recombination at *ade6* hotspots (41–43).

Particularly noteworthy is the preferential repair of hotspot DSBs with the sister chromatid, as shown by the greater abundance of IS HJs than IH HJs (Figure 5b). By measuring recombination in many intervals, with and without DSB hotspots, we observed crossover invariance, constant cM/kb whether or not the interval assayed contained a DSB hotspot (34). Our subsequent observations on IS and IH HJs and the genetic requirements for recombination at DSB hotspots and in DSB cold regions explained crossover invariance by partner choice for DSB repair: DSBs at hotspots are repaired primarily with the sister chromatid and give no genetic recombinants, whereas those in DSB cold regions are frequently, perhaps exclusively, repaired with the homolog and can give genetic recombinants (25). Our observations in this article fortify this conclusion, by showing that the IS:IH ratio is high at hotspots at 25°C, the condition for recombination assays, as well as at 34°C, the previous condition for HJ assays.

In our assays of HJs at 25°C, we observed a relatively stable DNA intermediate with the electrophoretic mobility expected for SEIs, likely equivalent to the D-loop formed by the initial DNA strand exchange (Figures 1 and 4a). This species has a smaller mass than the HJs, as expected for SEIs, and is at the position expected of Y-shaped DNA with a branch-point at the middle of the restriction fragment and where the DSBs are initiated. The appearance of these intermediates occurs after DNA replication has been completed, indicating they arise from DSB repair during meiotic recombination. Furthermore, during the time of replication, the abundance of branched DNA molecules with different masses along the Y-arc is nearly constant: the hybridization signal (with the central-region probe) of the larger molecules is nearly twice that of the smaller molecules (Figure 4a), as expected for uniformly located replication forks along the genome fragment assayed. In contrast, the molecules arising after replication is complete (after 6 h) have nearly the same mass, one that indicates a branch point restricted to the site of prominent DSBs, consistent with this species arising by strand invasion at that site. This species also disappeared more rapidly than HJs, consistent with its being a precursor to HJs. We previously observed a similar DNA intermediate that was most apparent at 34°C only after crosslinking the DNA with psoralen before extraction from cells (22). We infer that this species forms at both temperatures but is unstable at 34°C without crosslinking. Thermal instability is consistent with this species being an SEI. Further analysis of this species using the conditions used here may confirm this inference. More generally, having the same, optimal conditions for both genetic and physical analyses will lead to a more complete understanding of the mechanism of meiotic recombination.

## ACCESSION NUMBERS

Raw data in Figure 3, Supplementary Figures S1 and S2 are deposited in the NCBI Gene Expression Omnibus (GEO) as accession number GSE49961.

## SUPPLEMENTARY DATA

Supplementary Data are available at NAR Online, including [44,45].

## FUNDING

Funding for open access charge: United States of America National Institutes of Health grants [GM031693 and GM032194 to G.R.S.] and Austrian Science Fund (FWF) grants [P23609 and P21437 to J.G.]. The European Community’s Seventh Framework Programme (FP7/2007-2013) grant agreement number [PERG07-GA-2010-268167 to L.C.] and grant agreement number [PCIG11-GA-2012-322300 to J.G.].

*Conflict of interest statement*. None declared.

## Supplementary Material

Supplementary Data
