# Supplementary material for: DNA intermediates of meiotic recombination in synchronous S. pombe at optimal temperature
Source: Nucleic Acids Res. 2013 Oct 1;42(1):359–69. doi: 10.1093/nar/gkt861 (PMC3874177; doi:10.1093/nar/gkt861)
Supplement: Supplementary Data [file supp_42_1_359__index.html]

DNA intermediates of meiotic recombination in synchronous S. pombe at optimal temperature — DNA intermediates of meiotic recombination in synchronous S. pombe at optimal temperature — Supplementary Data 

# DNA intermediates of meiotic recombination in synchronous *S. pombe* at optimal temperature

## Supplementary Data

files

**Files in this Data Supplement:**

- Supplementary Data - pdf file
