# Supplementary material for: DNA intermediates of meiotic recombination in synchronous S. pombe at optimal temperature
Source: Nucleic Acids Res. 2013 Oct 1;42(1):359–69. doi: 10.1093/nar/gkt861 (PMC3874177; doi:10.1093/nar/gkt861)
Supplement: Supplementary Data [file supp_gkt861_nar-02281-a-2013-File008.pdf]

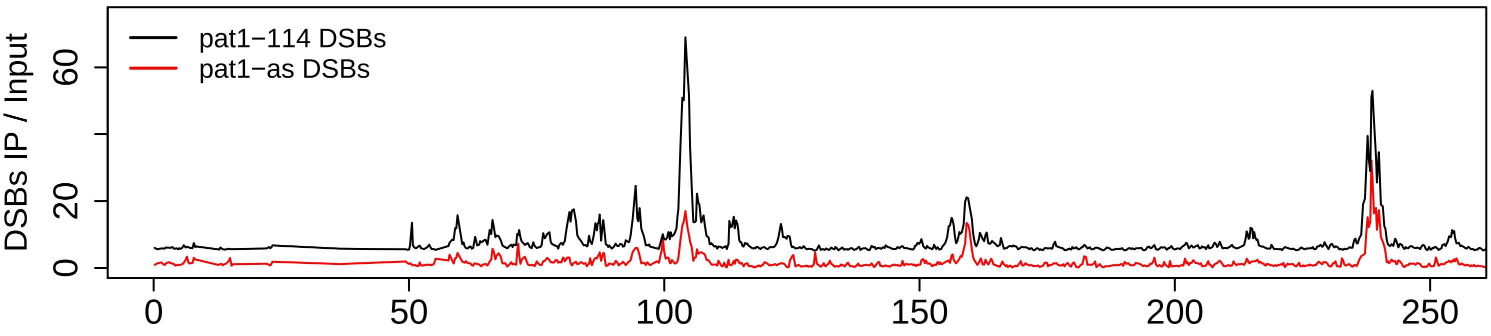
pat1−114 DSBs
pat1−as DSBs
DSBs IP / Input

Figure S1

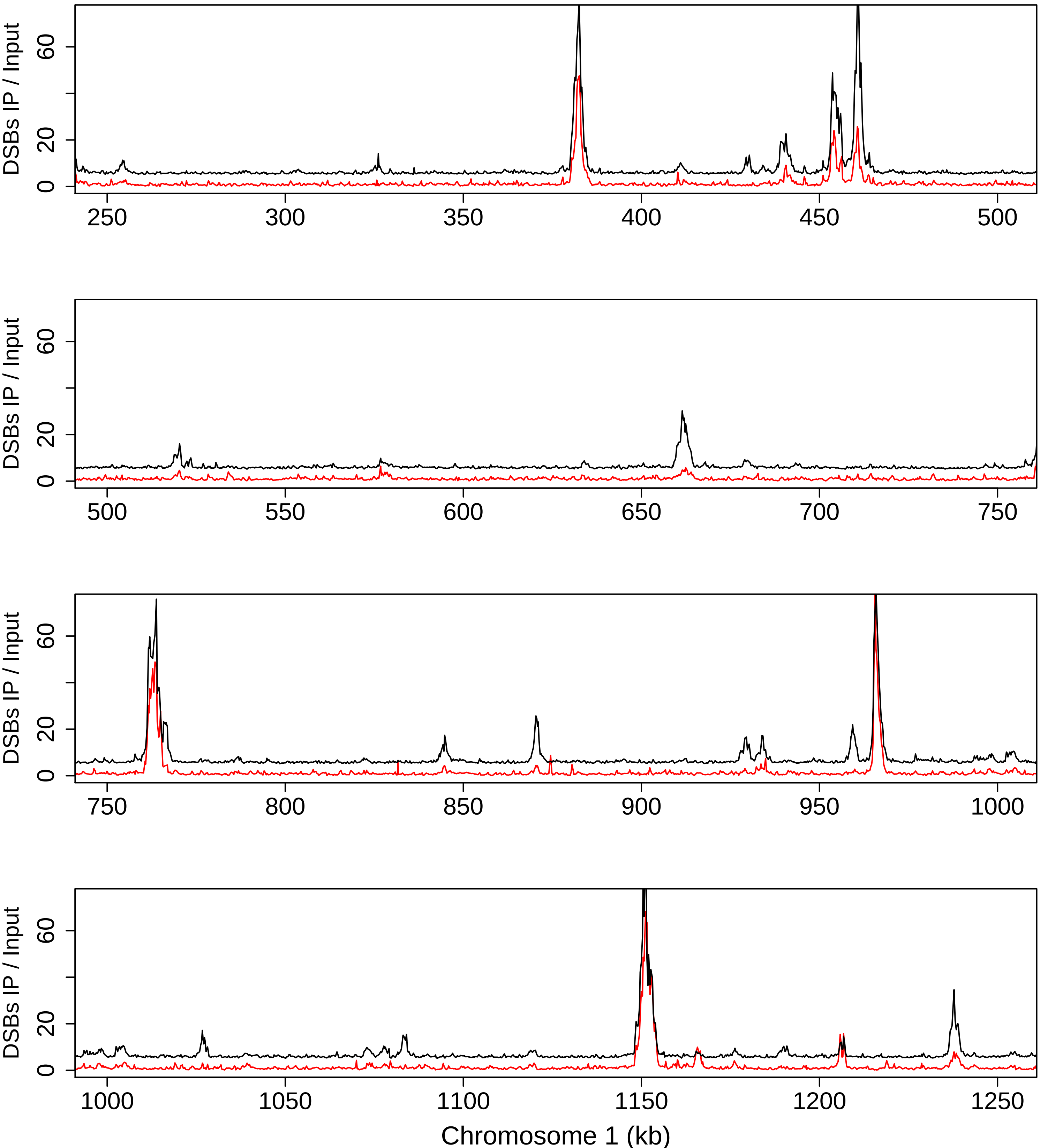
DSBs IP / Input
Chromosome 1 (kb)

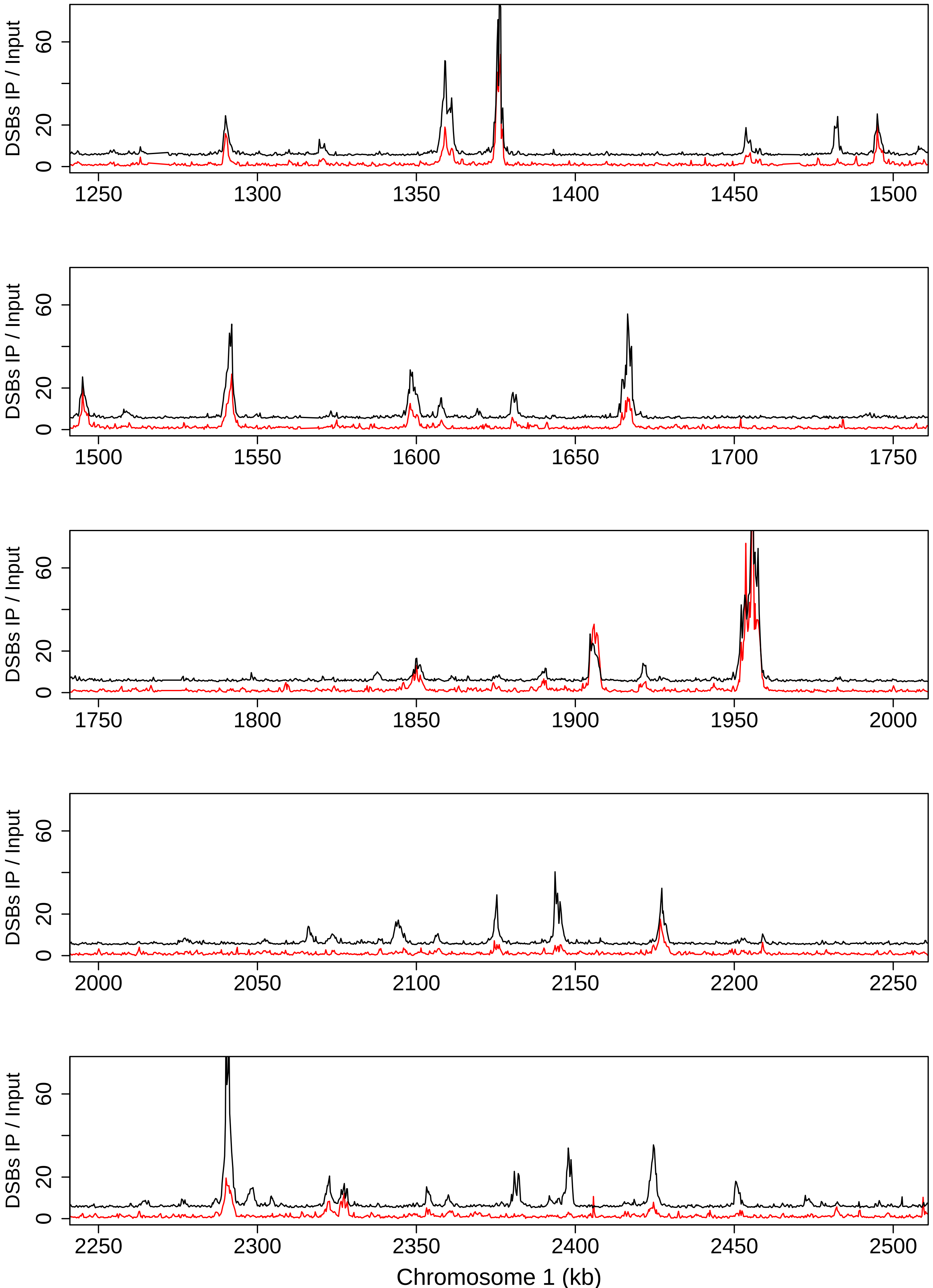

DSBs IP / Input
0
20
60
1250
1300
1350
1400
1450
1500
1550
1600
1650
1700
1750
1800
1850
1900
1950
2000
2050
2100
2150
2200
2250
2300
2350
2400
2450
2500
Chromosome 1 (kb)

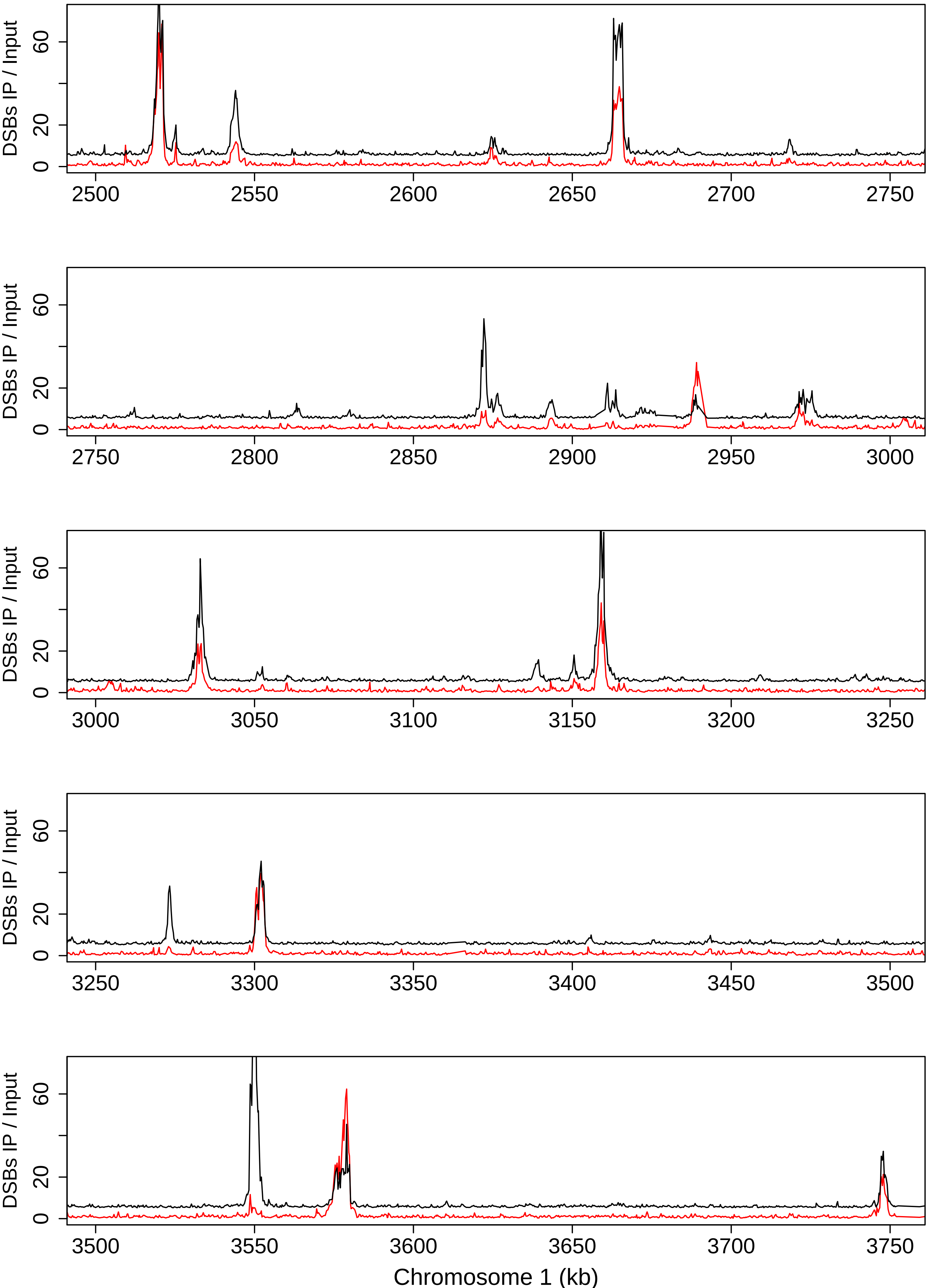

DSBs IP / Input
0
20
60
2500
2550
2600
2650
2700
2750
2800
2850
2900
2950
3000
3050
3100
3150
3200
3250
3300
3350
3400
3450
3500
3550
3600
3650
3700
3750
Chromosome 1 (kb)

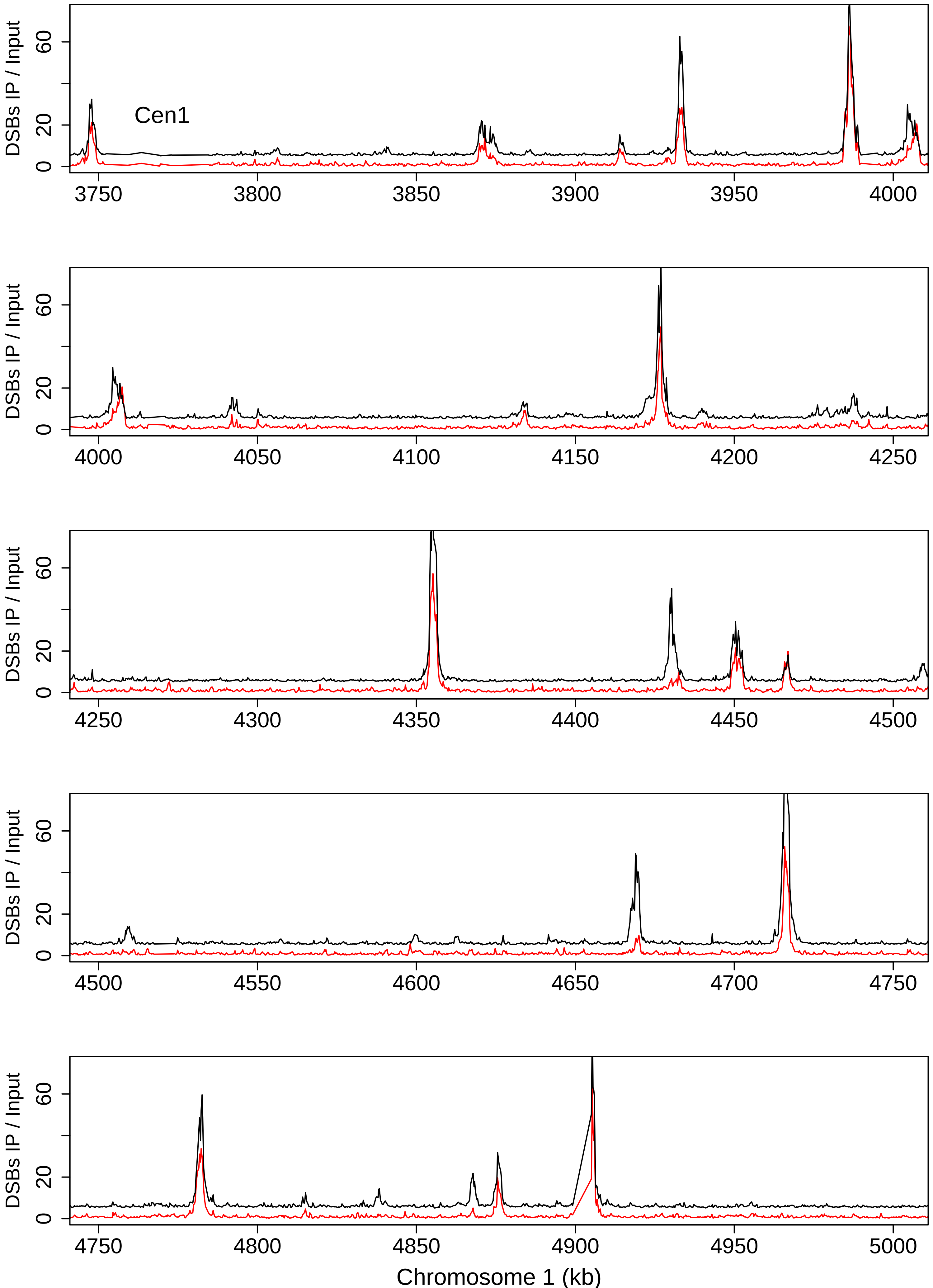

Cen1
DSBs IP / Input
0
20
60
3750
3800
3850
3900
3950
4000
4050
4100
4150
4200
4250
4300
4350
4400
4450
4500
4550
4600
4650
4700
4750
4800
4850
4900
4950
5000
Chromosome 1 (kb)

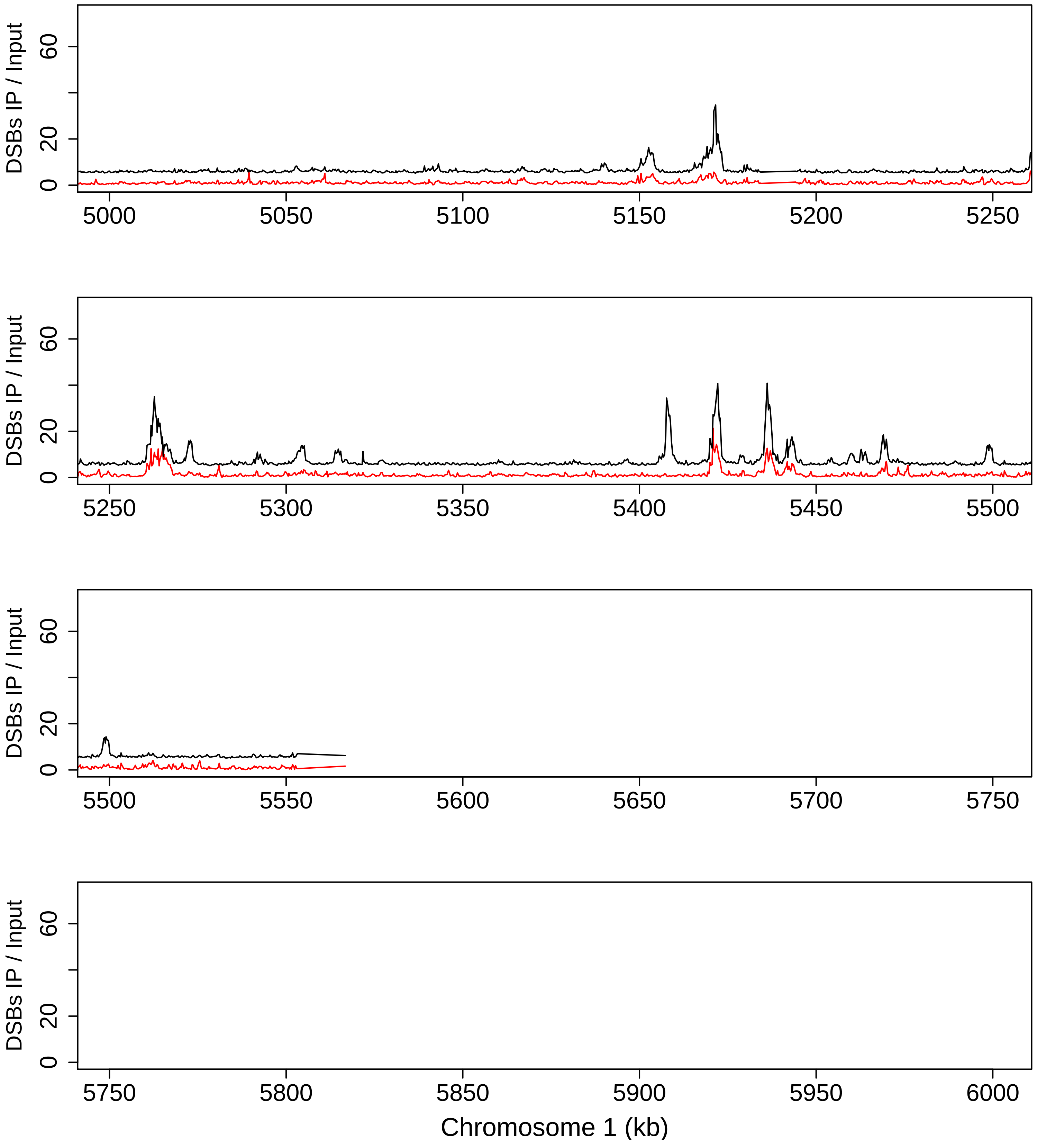
DSBs IP / Input
0
20
60
5000
5050
5100
5150
5200
5250
5300
5350
5400
5450
5500
5550
5600
5650
5700
5750
5800
5850
5900
5950
6000
Chromosome 1 (kb)

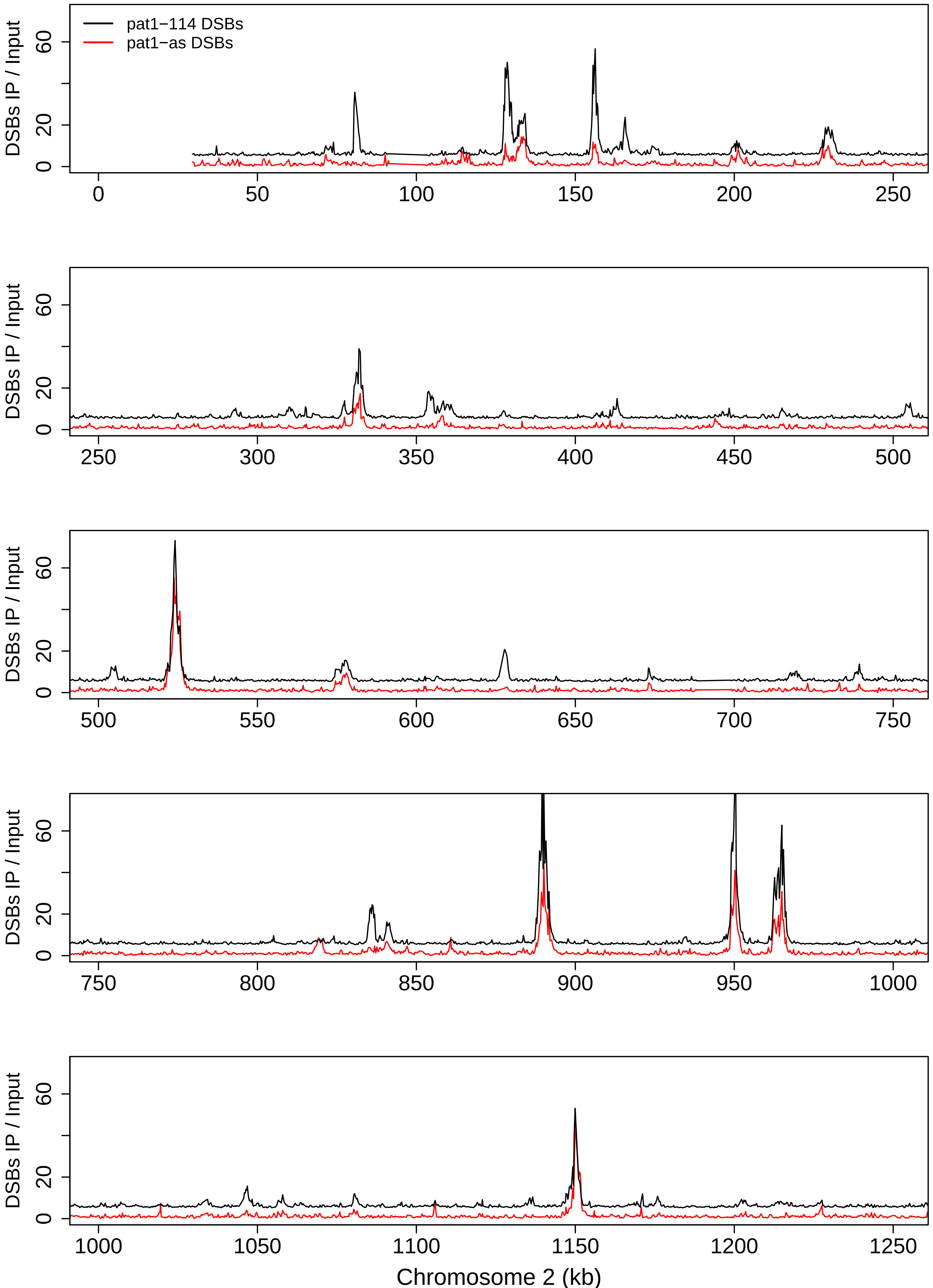

pat1−114 DSBs
pat1−as DSBs
DSBs IP / Input
0
20
60
0
50
100
150
200
250
300
350
400
450
500
550
600
650
700
750
800
850
900
950
1000
1050
1100
1150
1200
1250
Chromosome 2 (kb)

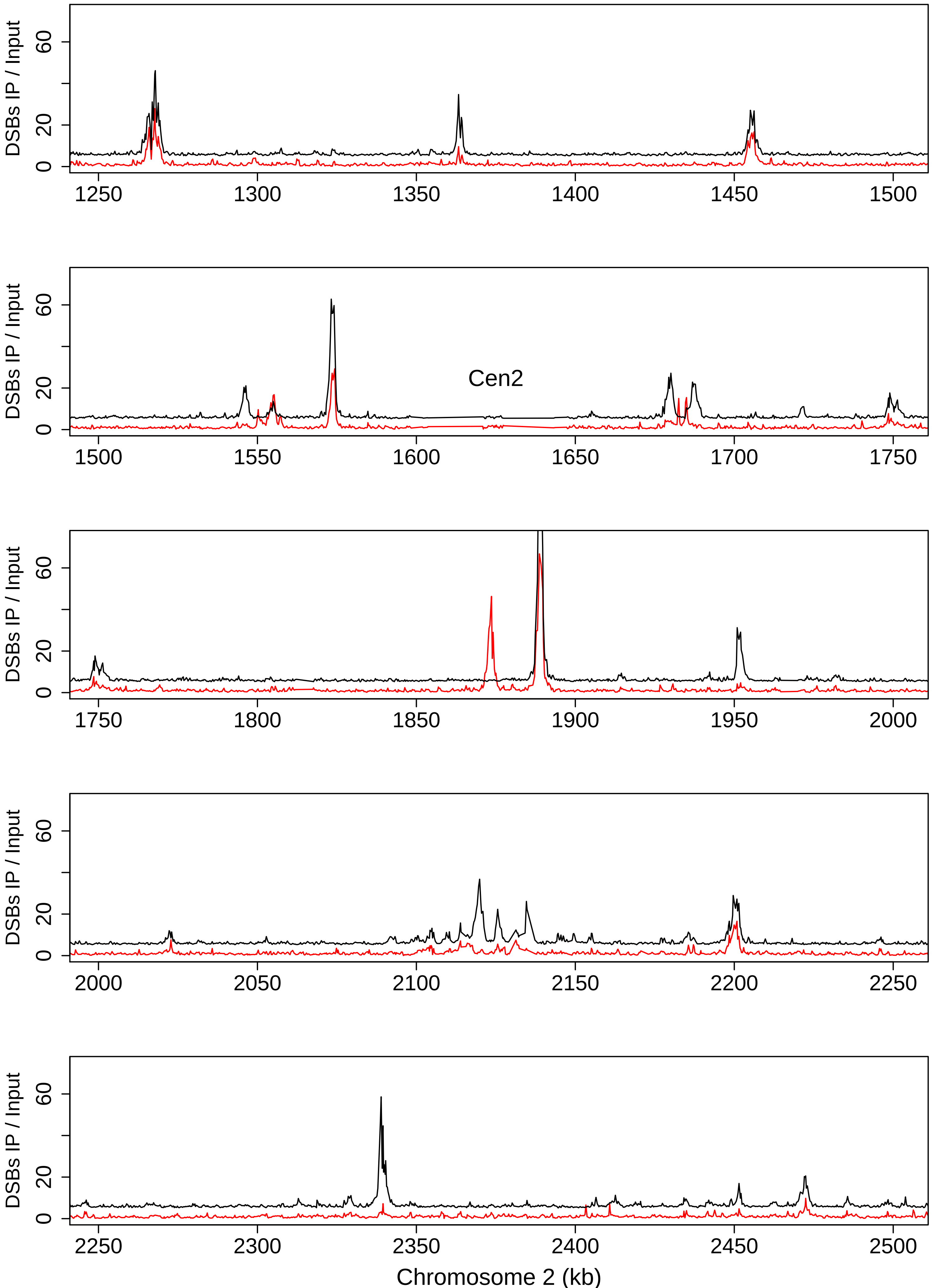
DSBs IP / Input
0
20
60
1250
1300
1350
1400
1450
1500
Cen2
1550
1600
1650
1700
1750
1800
1850
1900
1950
2000
2050
2100
2150
2200
2250
2300
2350
2400
2450
2500
Chromosome 2 (kb)

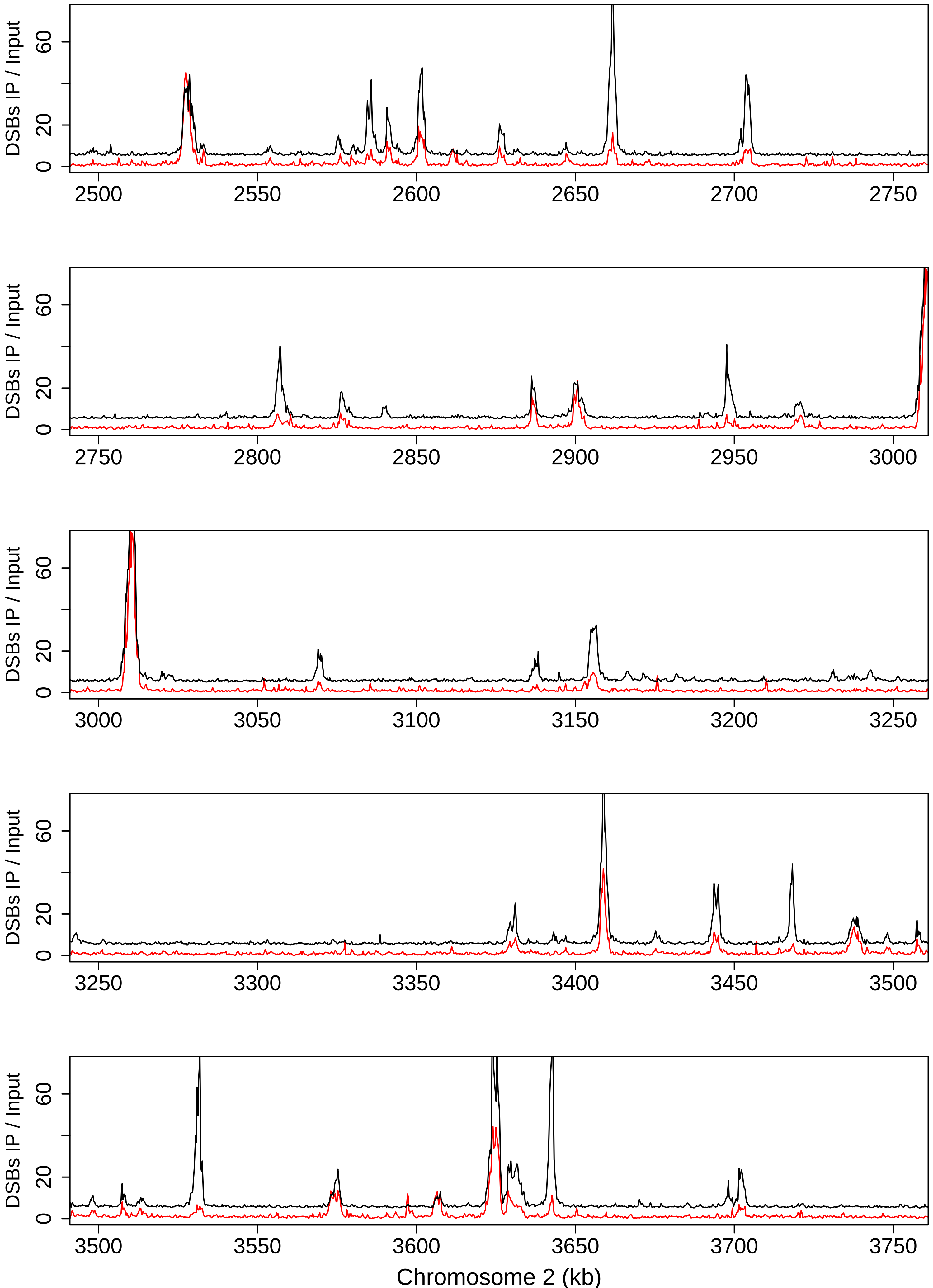

DSBs IP / Input
0
20
60
2500
2550
2600
2650
2700
2750
2800
2850
2900
2950
3000
3050
3100
3150
3200
3250
3300
3350
3400
3450
3500
3550
3600
3650
3700
Chromosome 2 (kb)

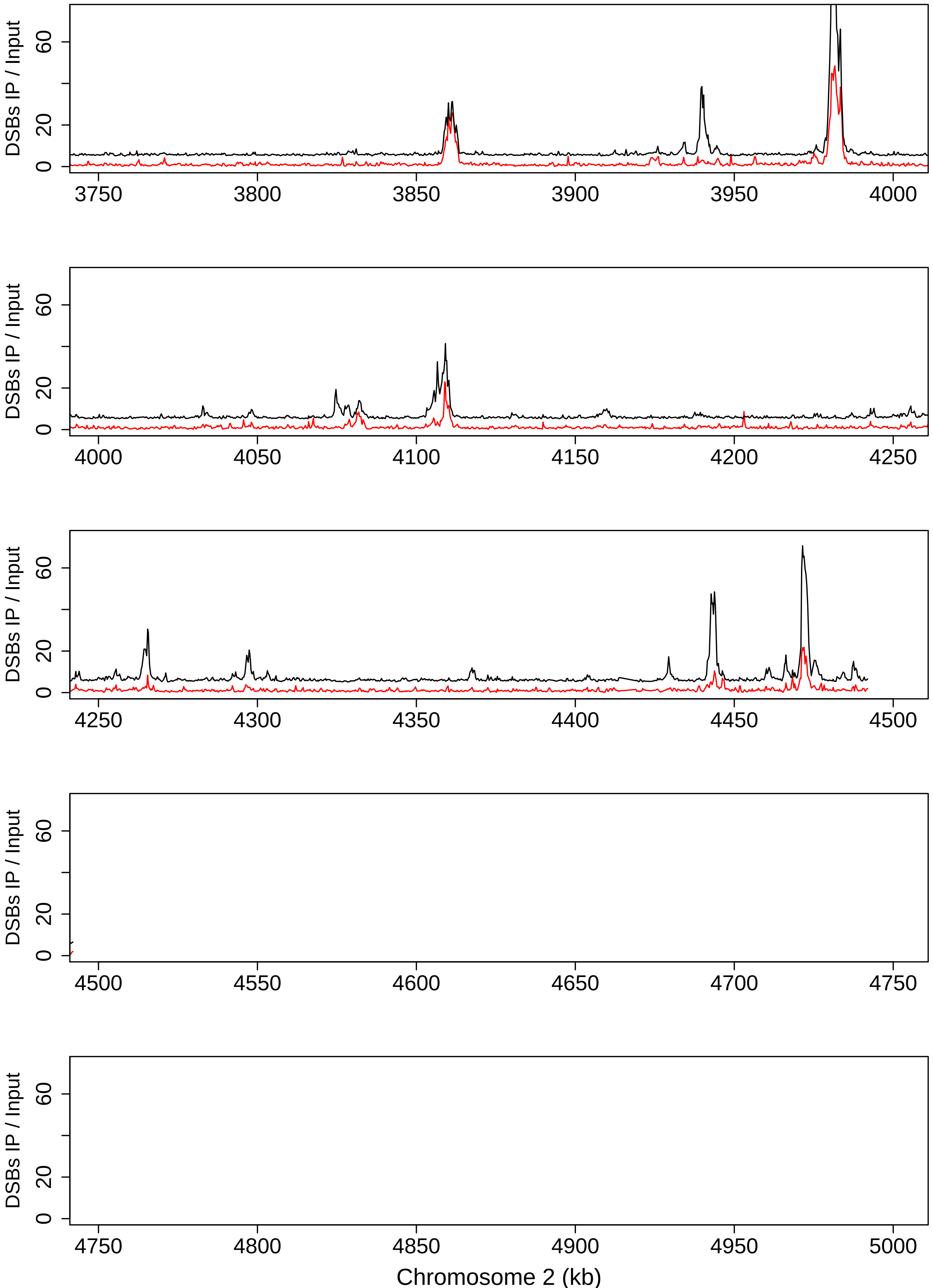
DSBs IP / Input
0
20
60
3750
3800
3850
3900
3950
4000
4050
4100
4150
4200
4250
4300
4350
4400
4450
4500
4550
4600
4650
4700
4750
4800
4850
4900
4950
5000
Chromosome 2 (kb)

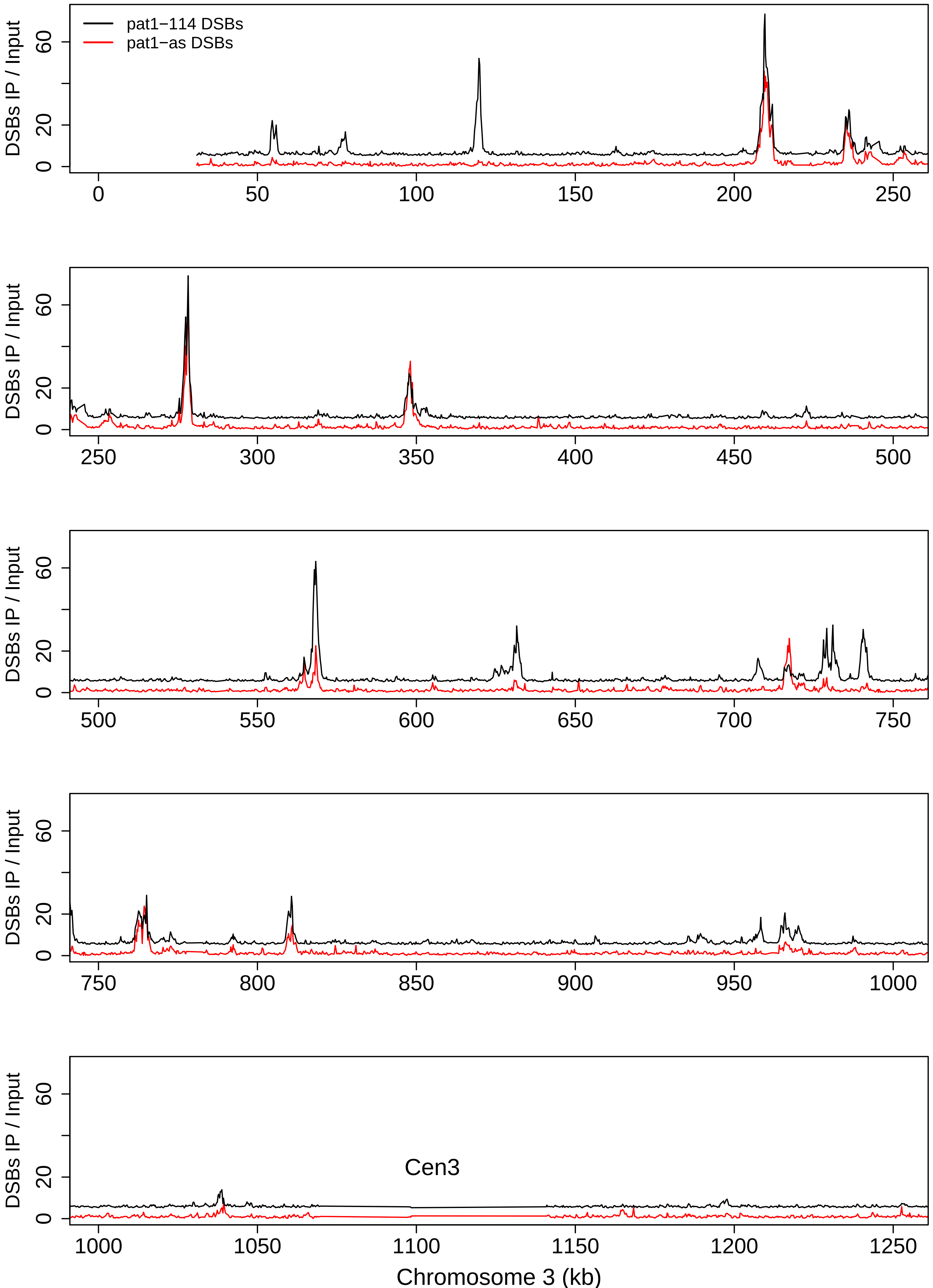
pat1−114 DSBs
pat1−as DSBs
DSBs IP / Input
Cen3
Chromosome 3 (kb)

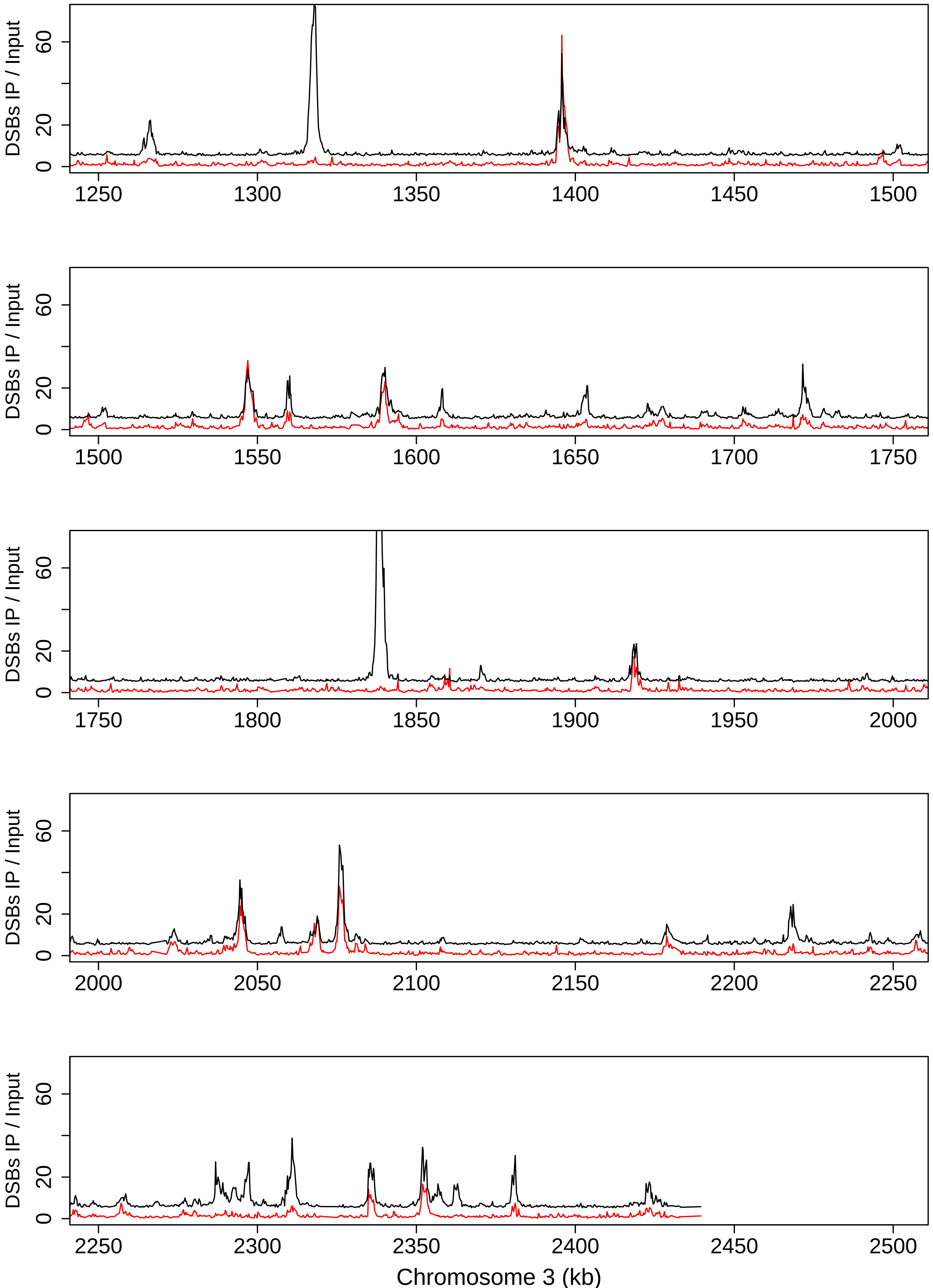
DSBs IP / Input
0
20
60
1250
1300
1350
1400
1450
1500
1550
1600
1650
1700
1750
1800
1850
1900
1950
2000
2050
2100
2150
2200
2250
2300
2350
2400
2450
2500
Chromosome 3 (kb)

Figure S2

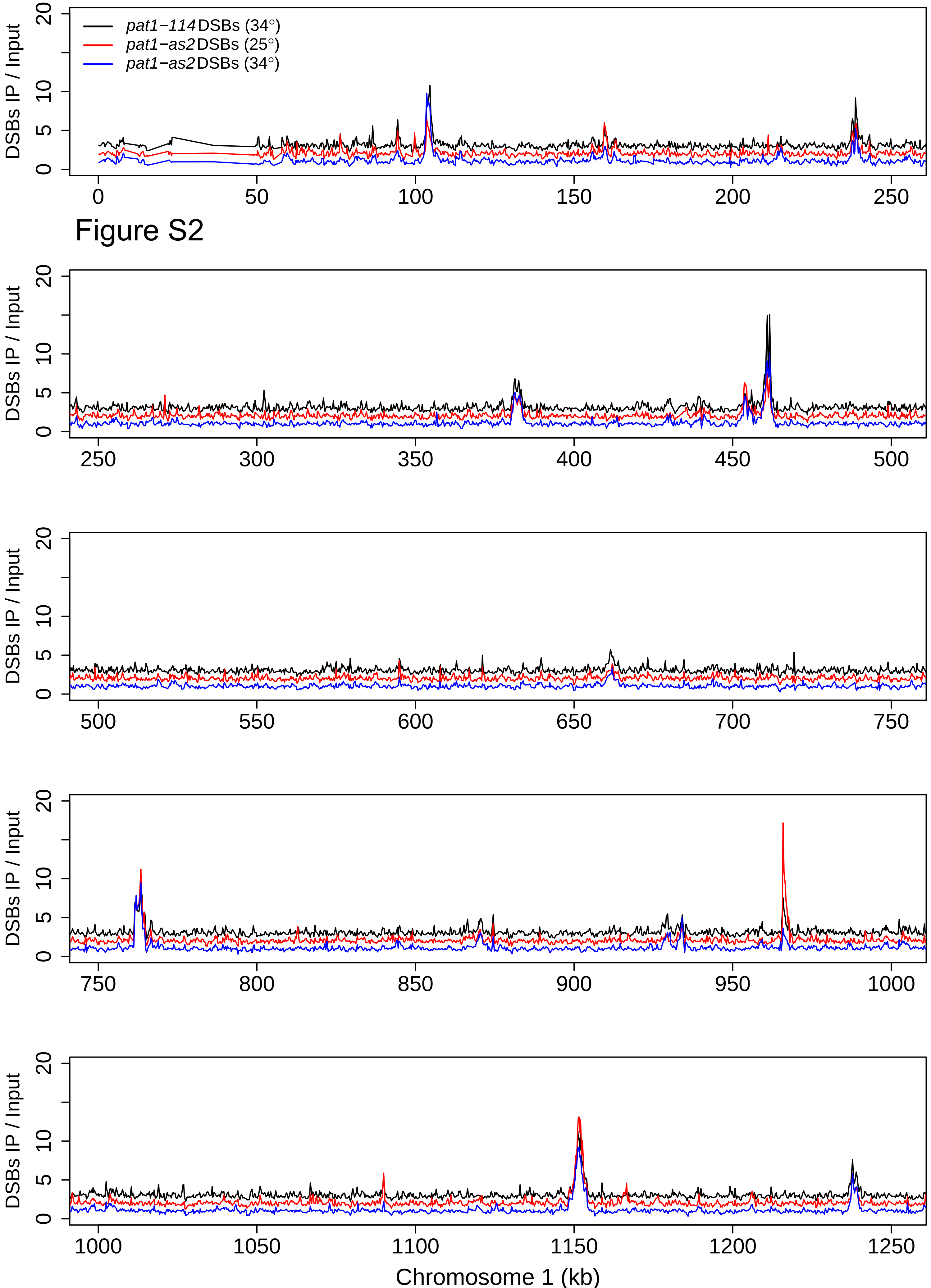

pat1−114 DSBs (34°)
pat1−as2 DSBs (25°)
pat1−as2 DSBs (34°)
DSBs IP / Input
Chromosome 1 (kb)

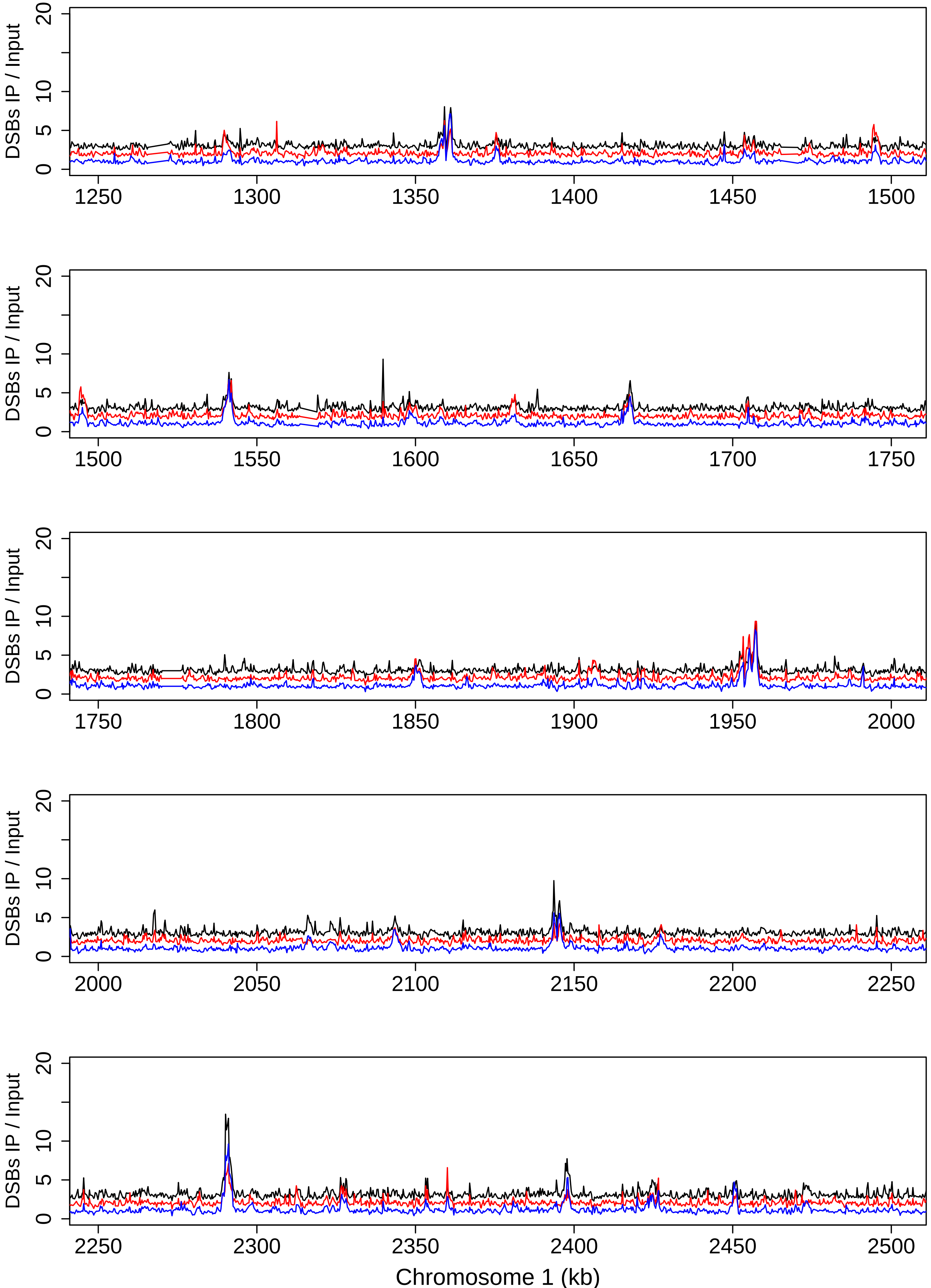

DSBs IP / Input
0
5
10
20
1250
1300
1350
1400
1450
1500
1550
1600
1650
1700
1750
1800
1850
1900
1950
2000
2050
2100
2150
2200
2250
2300
2350
2400
2450
2500
Chromosome 1 (kb)

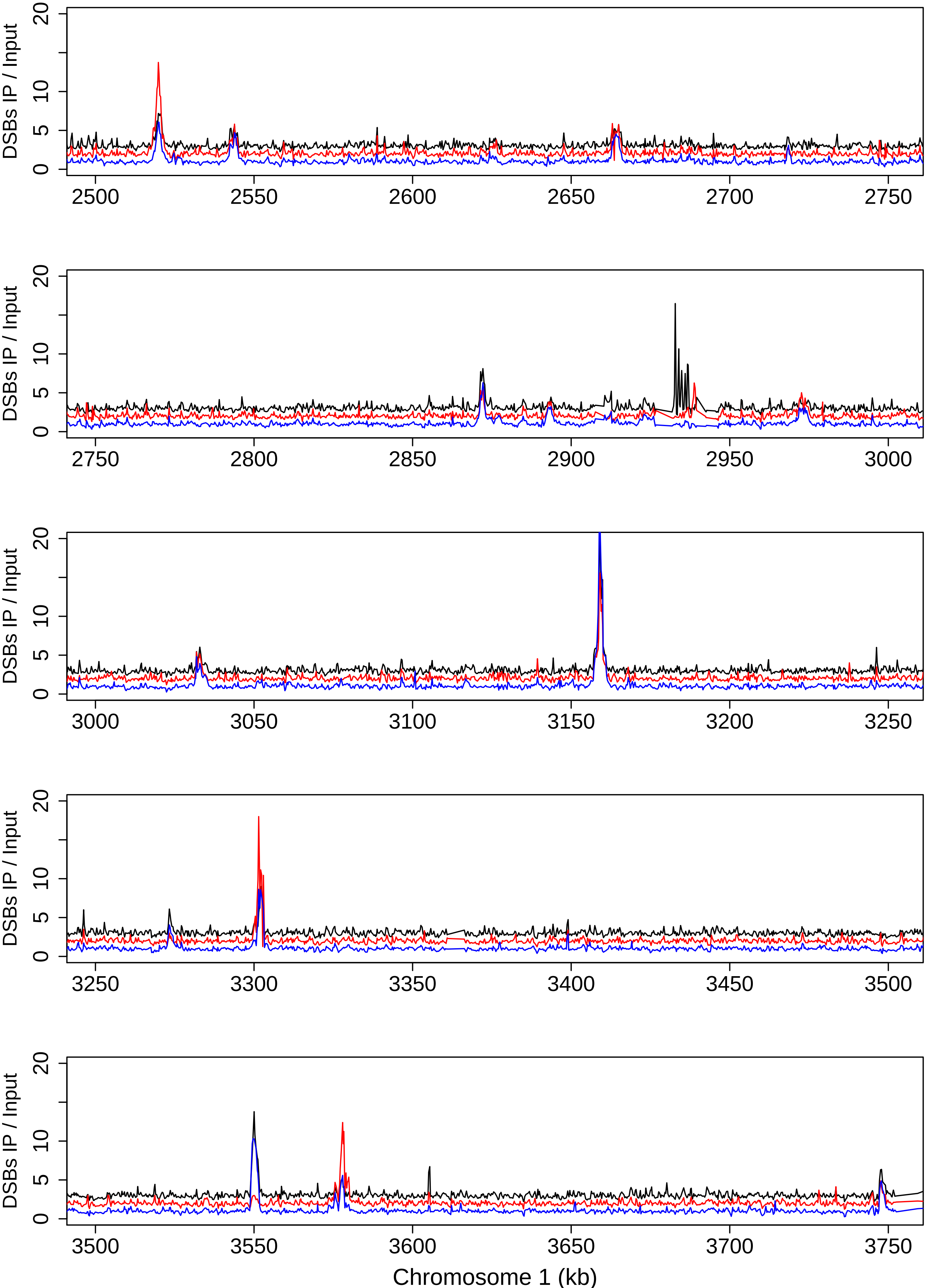
DSBs IP / Input
0
5
10
20
2500
2550
2600
2650
2700
2750
2800
2850
2900
2950
3000
3050
3100
3150
3200
3250
3300
3350
3400
3450
3500
3550
3600
3650
3700
3750
Chromosome 1 (kb)

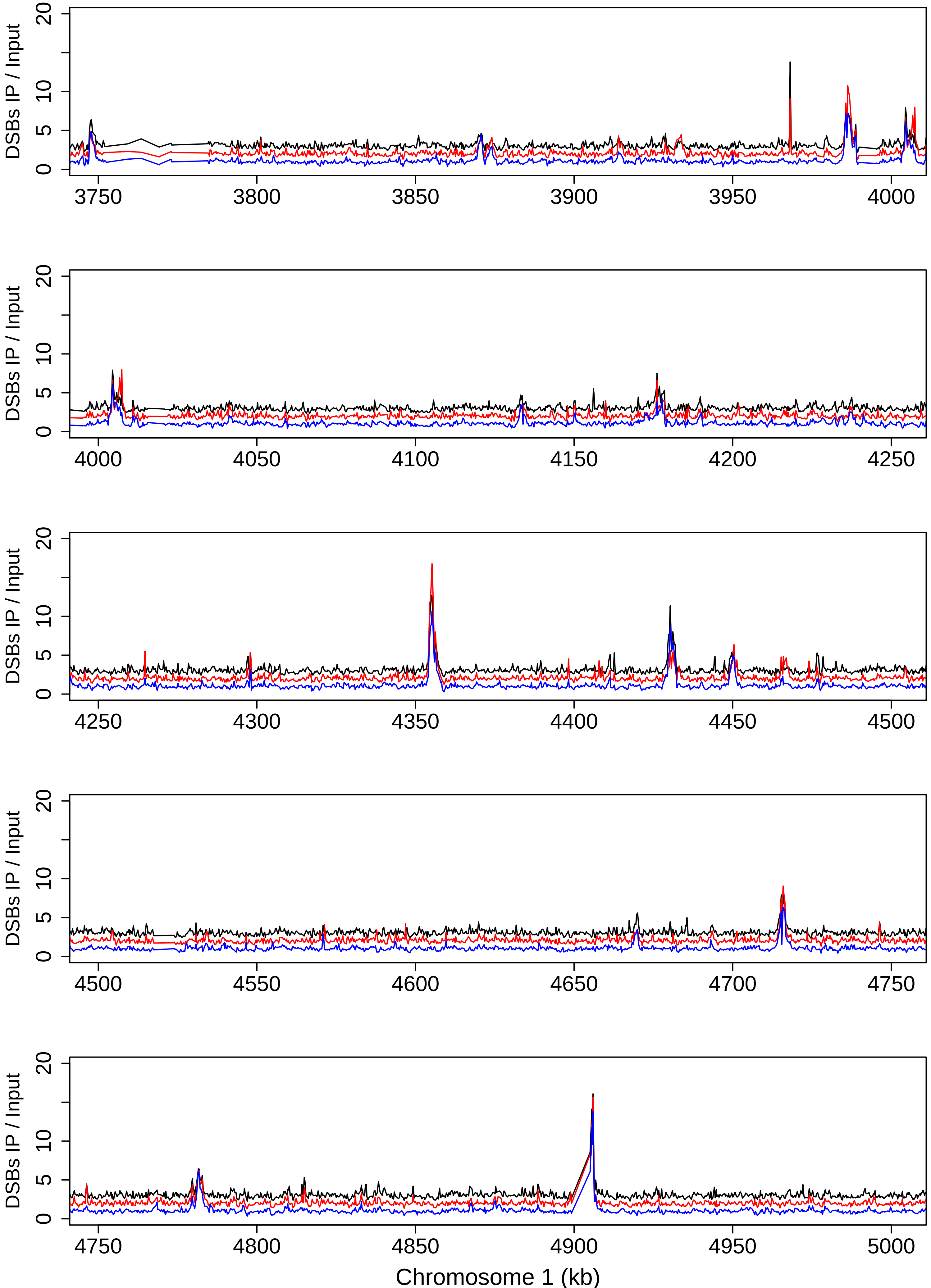

DSBs IP / Input
0
5
10
20
3750
3800
3850
3900
3950
4000
4050
4100
4150
4200
4250
4300
4350
4400
4450
4500
4550
4600
4650
4700
4750
4800
4850
4900
4950
5000
Chromosome 1 (kb)

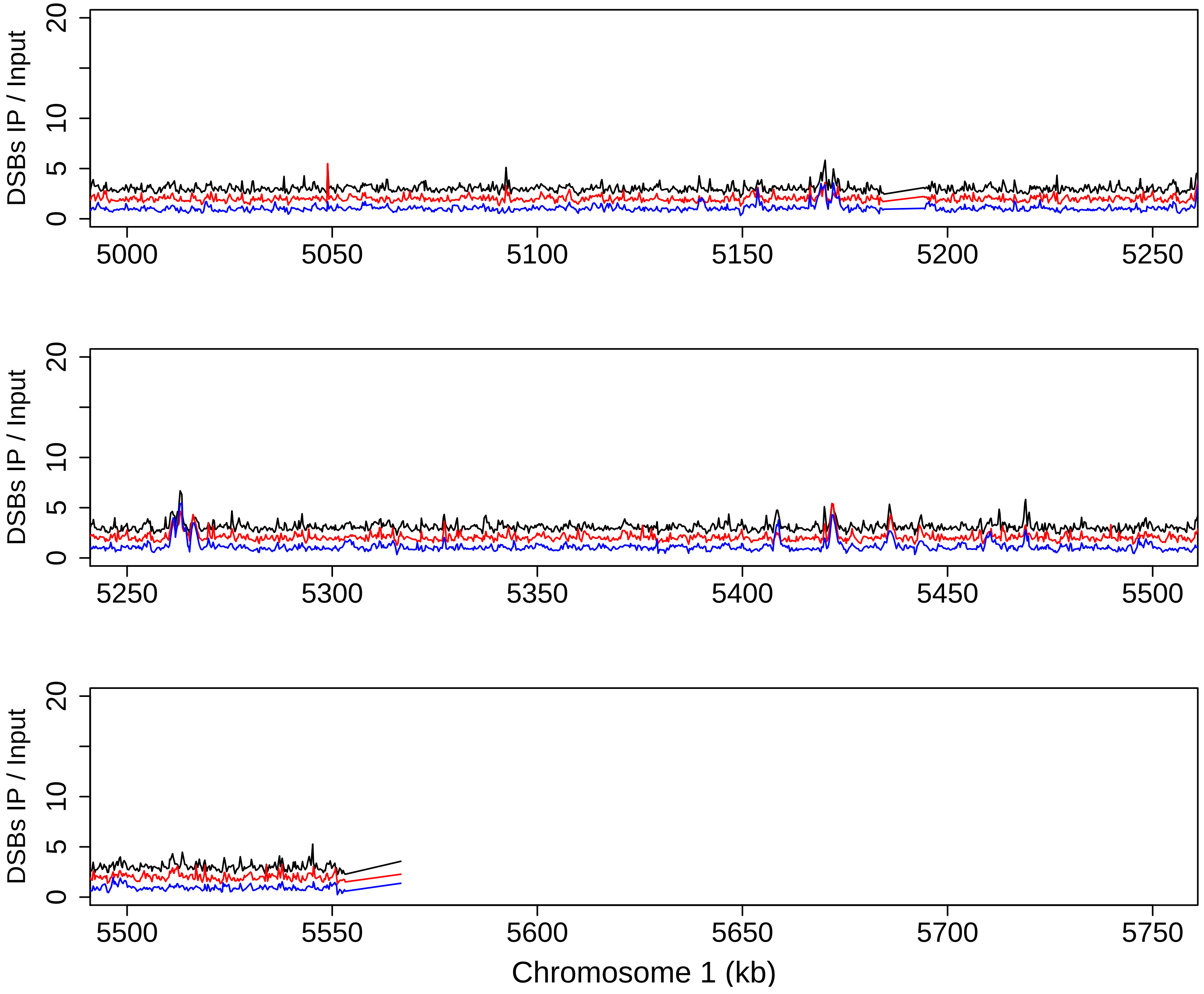

DSBs IP / Input
0
5
10
20
5000
5050
5100
5150
5200
5250
5300
5350
5400
5450
5500
5550
5600
5650
5700
5750
Chromosome 1 (kb)

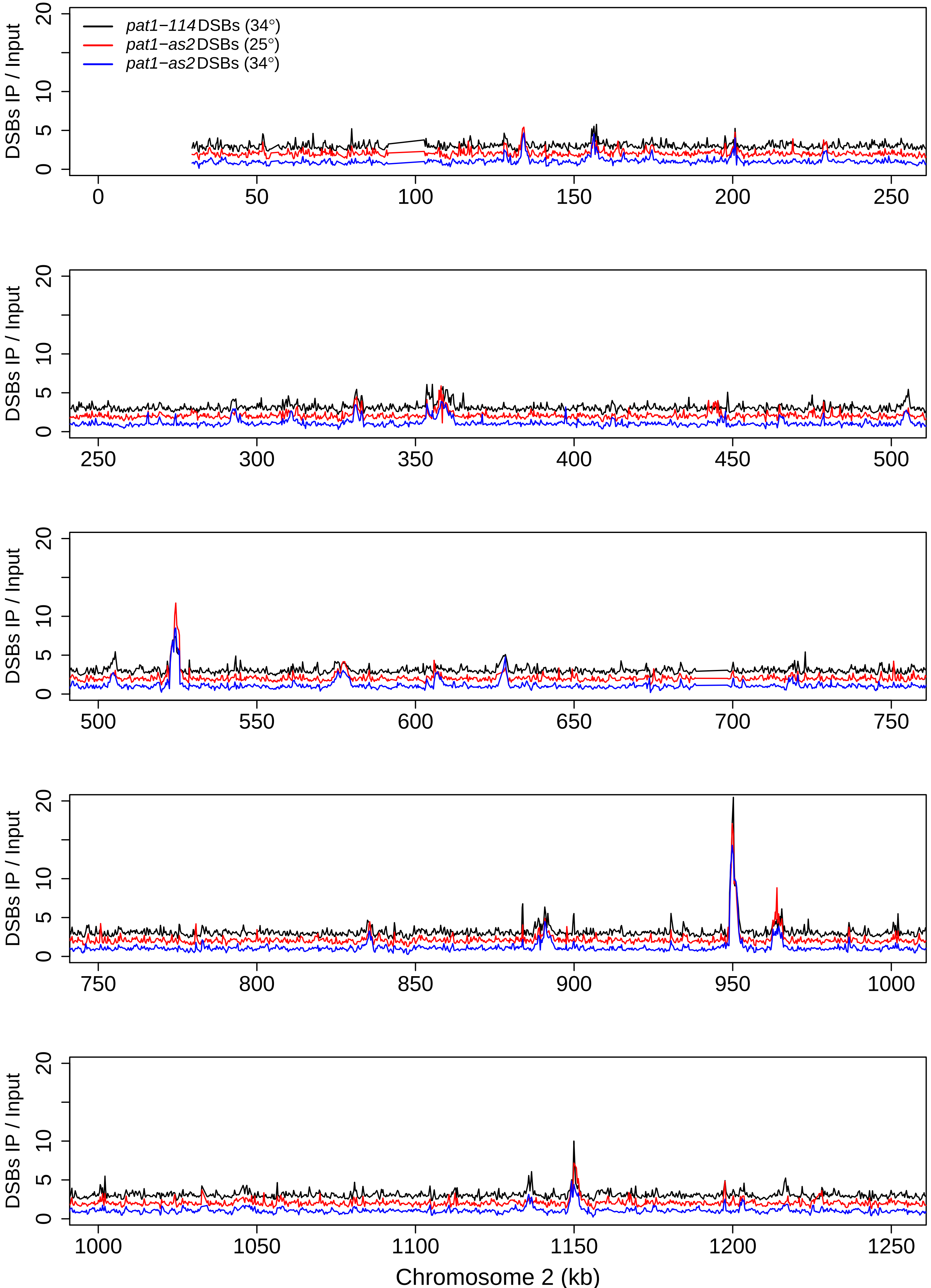

pat1−114 DSBs (34°)
pat1−as2 DSBs (25°)
pat1−as2 DSBs (34°)
DSBs IP / Input
Chromosome 2 (kb)

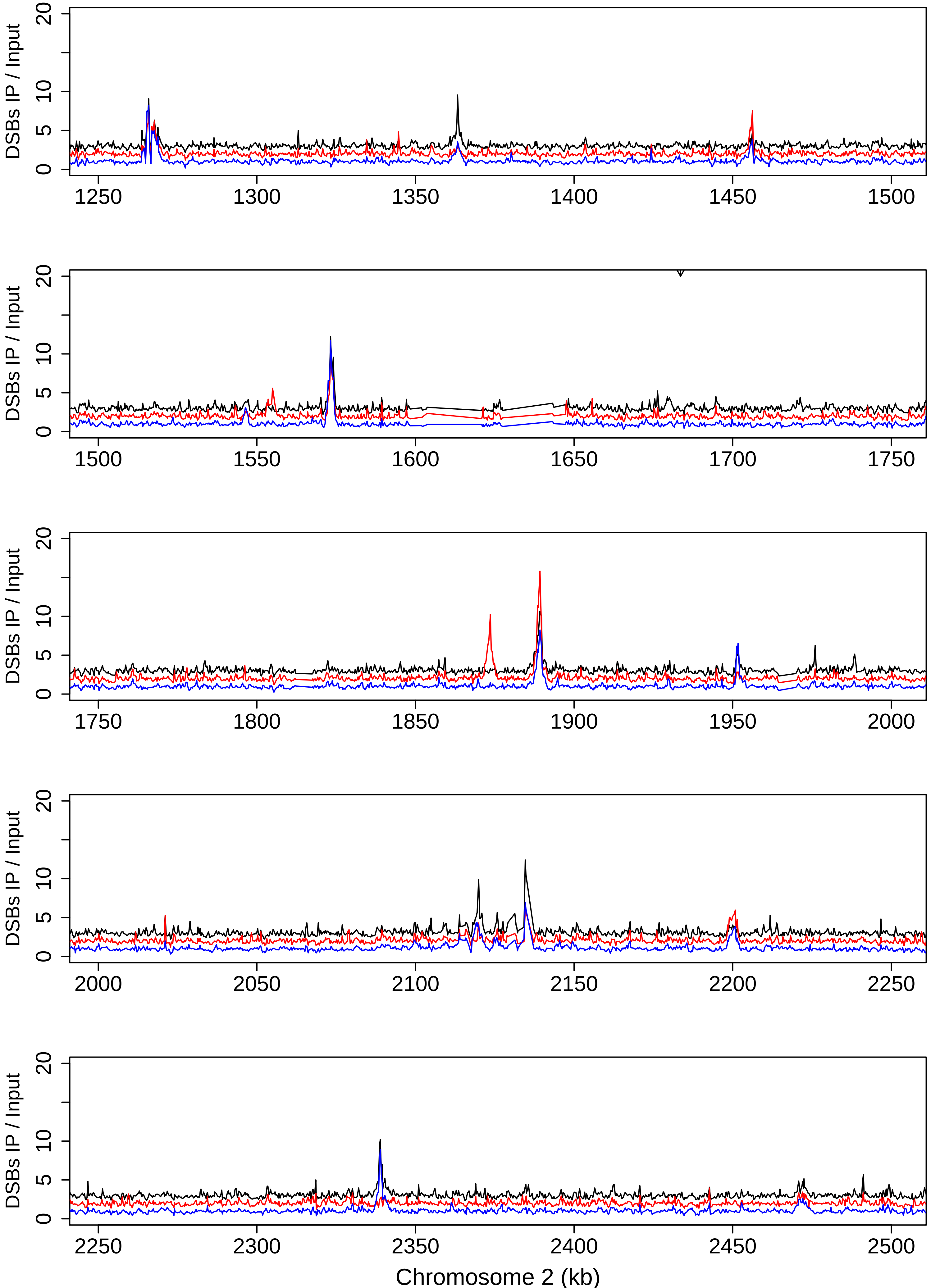

DSBs IP / Input
0
5
10
20
1250
1300
1350
1400
1450
1500
1550
1600
1650
1700
1750
1800
1850
1900
1950
2000
2050
2100
2150
2200
2250
2300
2350
2400
2450
2500
Chromosome 2 (kb)

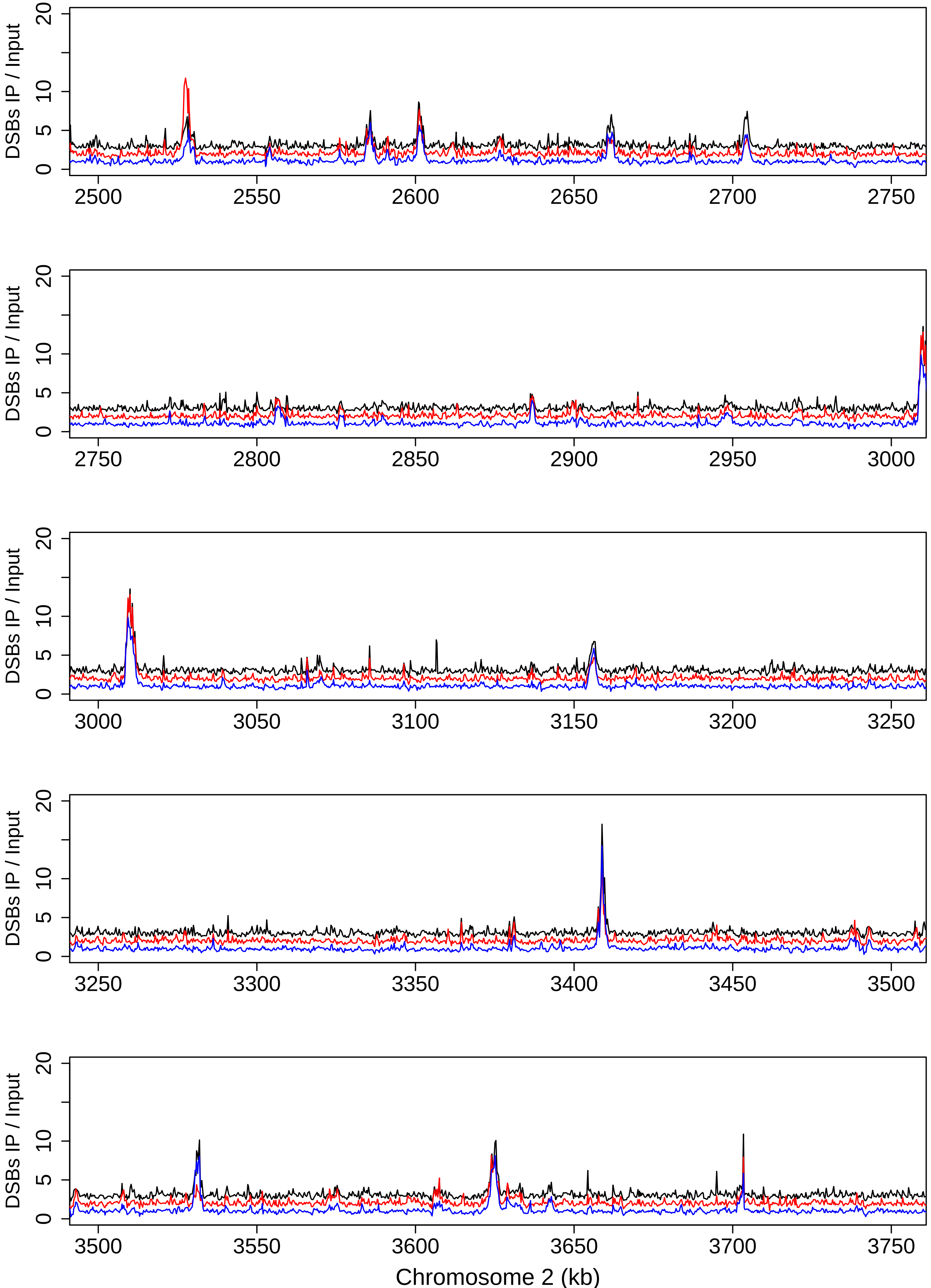

DSBs IP / Input
0
5
10
20
2500
2550
2600
2650
2700
2750
2800
2850
2900
2950
3000
3050
3100
3150
3200
3250
3300
3350
3400
3450
3500
3550
3600
3650
3700
3750
Chromosome 2 (kb)

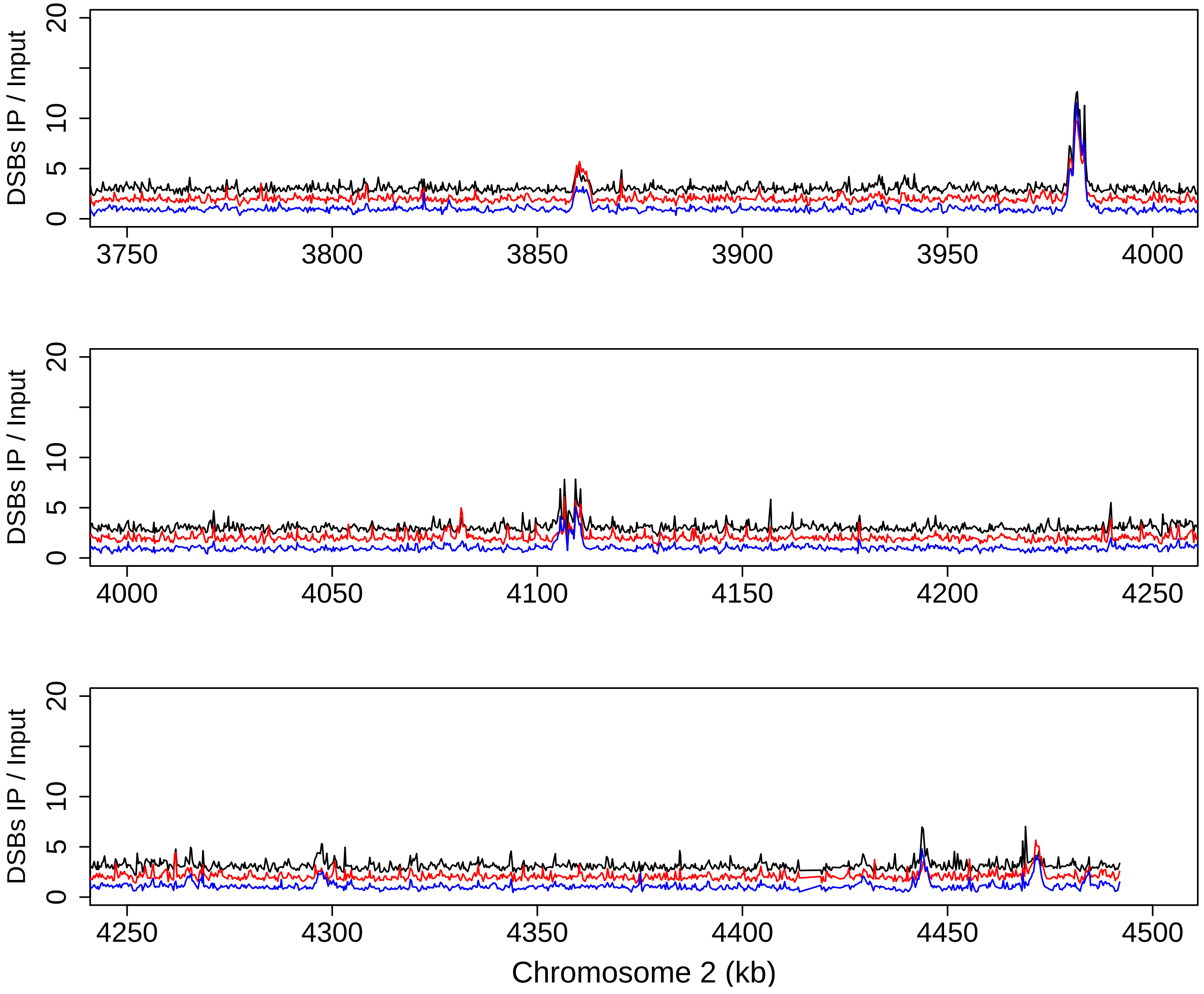
DSBs IP / Input
0
5
10
20
3750
3800
3850
3900
3950
4000
DSBs IP / Input
0
5
10
20
4000
4050
4100
4150
4200
4250
DSBs IP / Input
0
5
10
20
4250
4300
4350
4400
4450
4500
Chromosome 2 (kb)

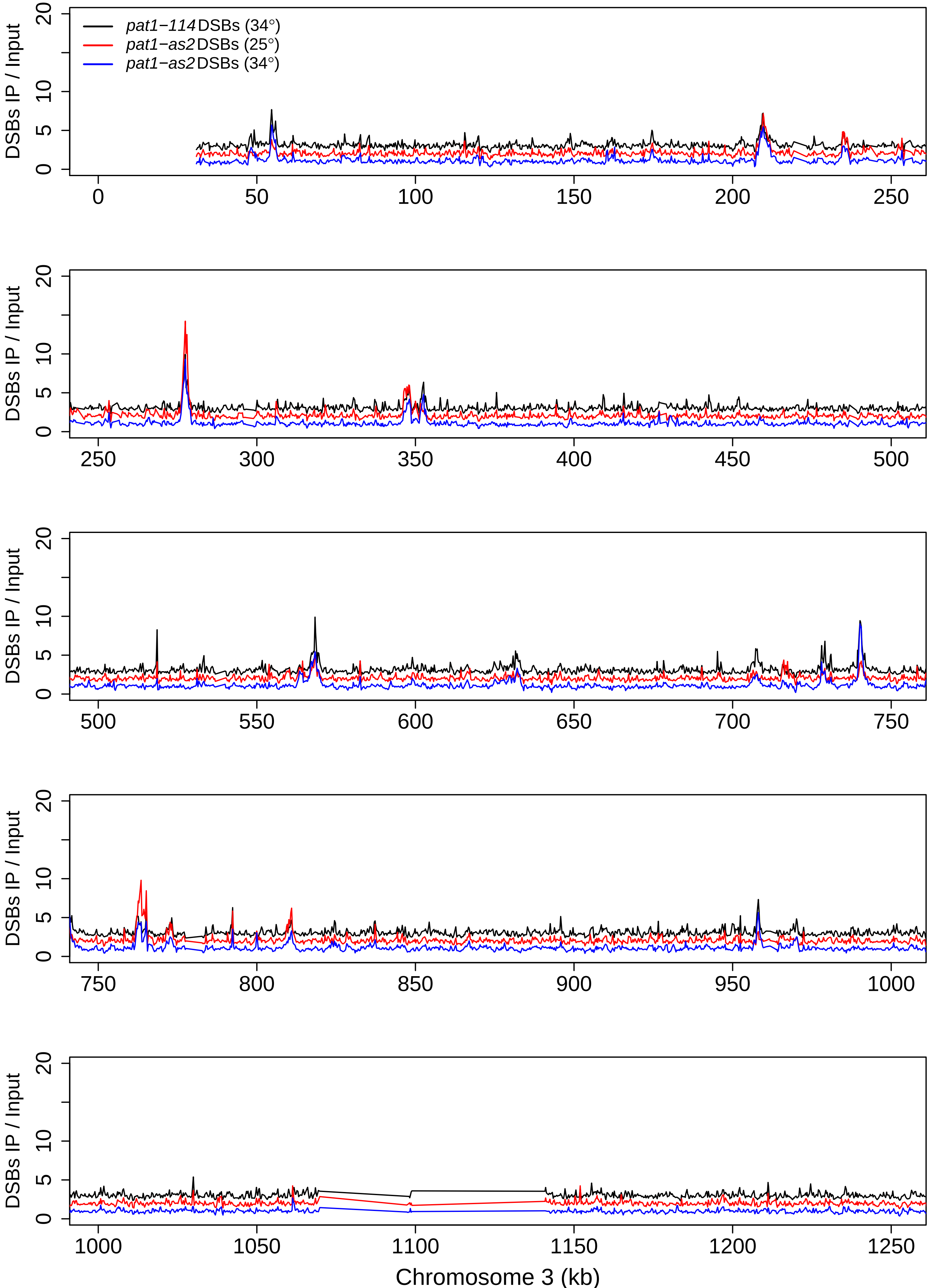
pat1−114 DSBs (34°)
pat1−as2 DSBs (25°)
pat1−as2 DSBs (34°)
DSBs IP / Input
Chromosome 3 (kb)

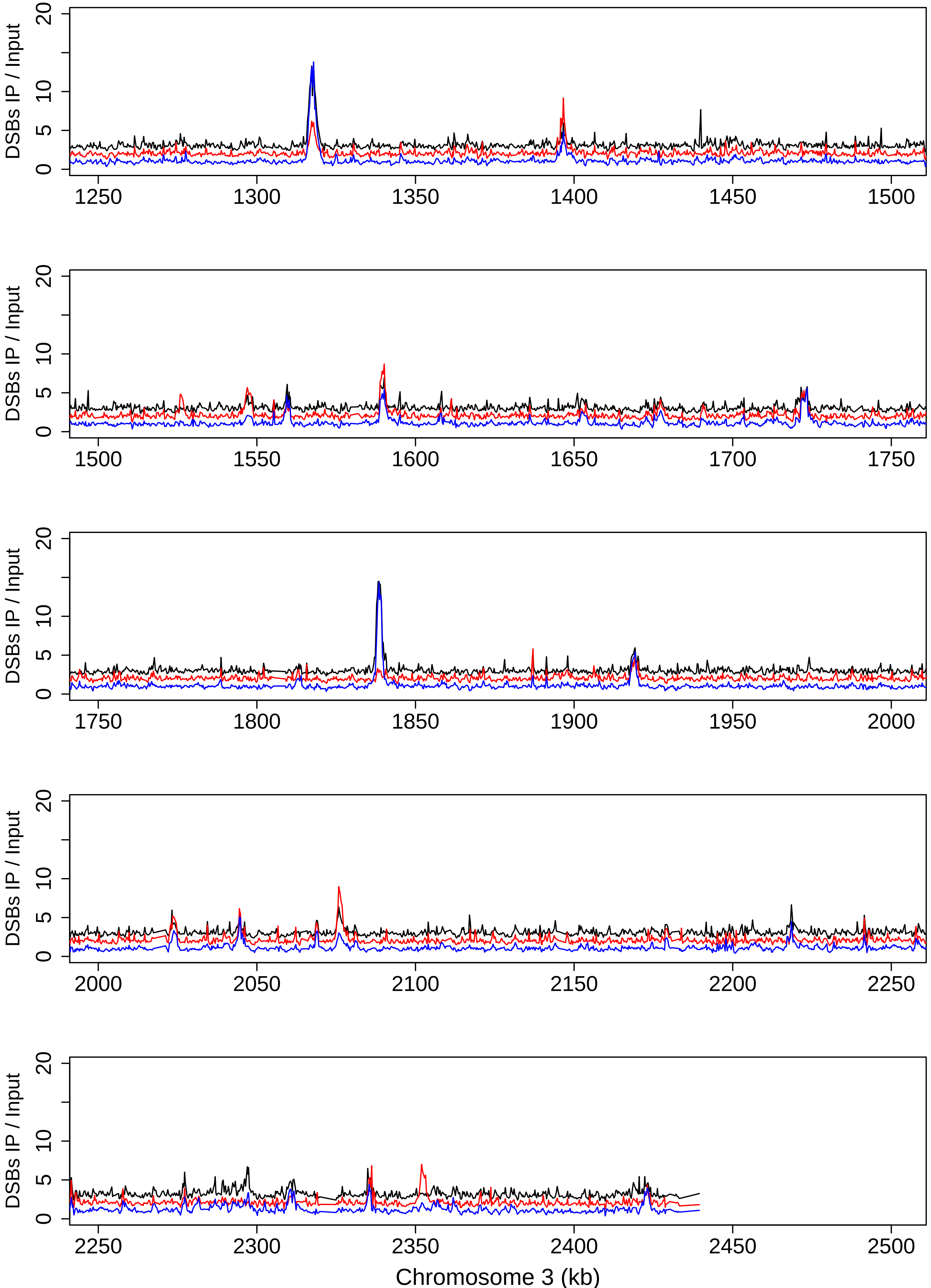
DSBs IP / Input
0
5
10
20
1250
1300
1350
1400
1450
1500
1550
1600
1650
1700
1750
1800
1850
1900
1950
2000
2050
2100
2150
2200
2250
2300
2350
2400
2450
2500
Chromosome 3 (kb)

Figure S3

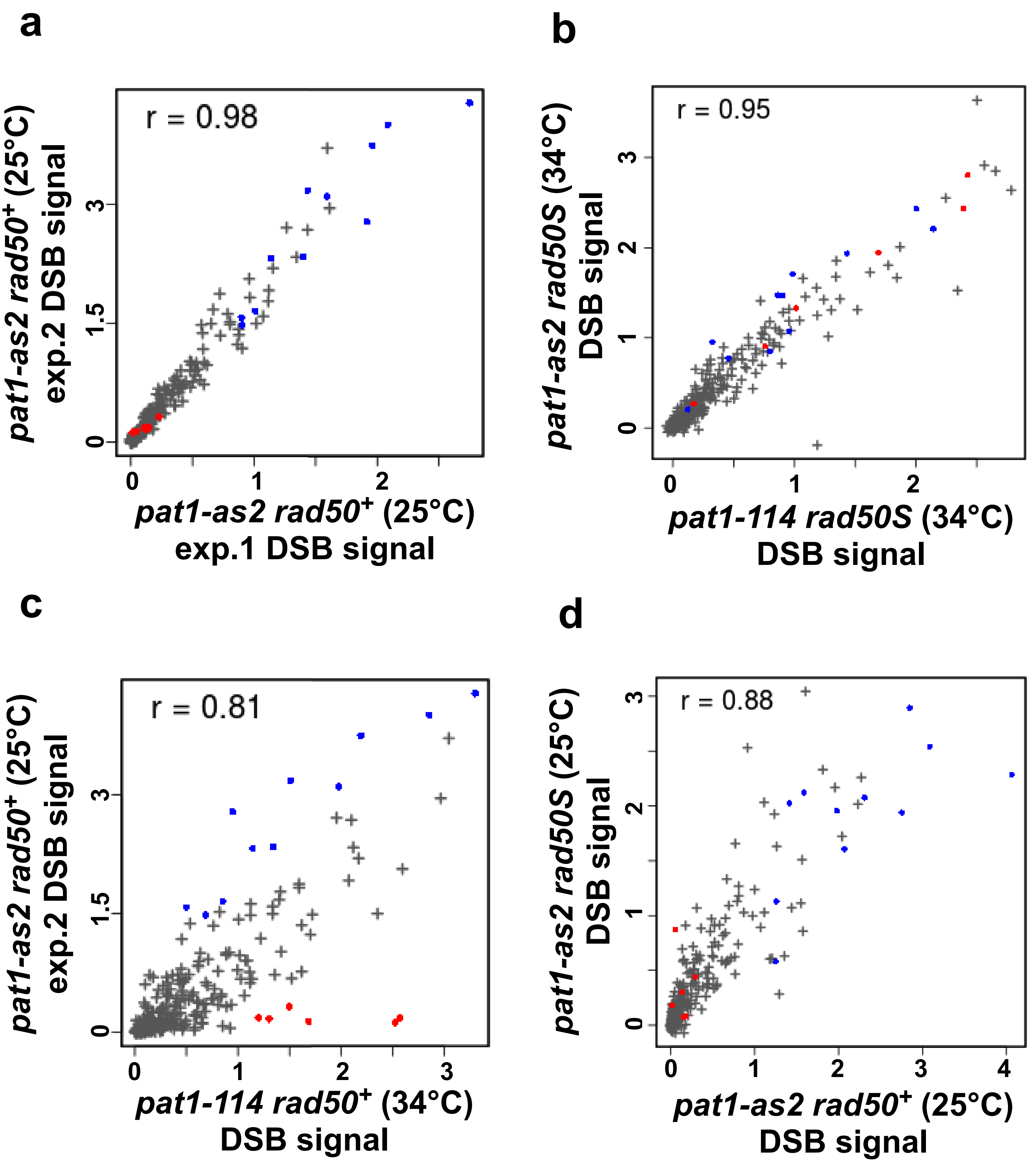
a
r = 0.98
pat1-as2 rad50+ (25°C) exp.2 DSB signal
pat1-as2 rad50+ (25°C) exp.1 DSB signal
0
1.5
3
0
1
2
b
r = 0.95
pat1-as2 rad50S (34°C) DSB signal
pat1-114 rad50S (34°C) DSB signal
0
1
2
3
0
1
2
c
r = 0.81
pat1-as2 rad50+ (25°C) exp.2 DSB signal
pat1-114 rad50+ (34°C) DSB signal
0
1.5
3
0
1
2
3
d
r = 0.88
pat1-as2 rad50S (25°C) DSB signal
pat1-as2 rad50+ (25°C) DSB signal
0
1
2
3
0
1
2
3
4

Figure S4

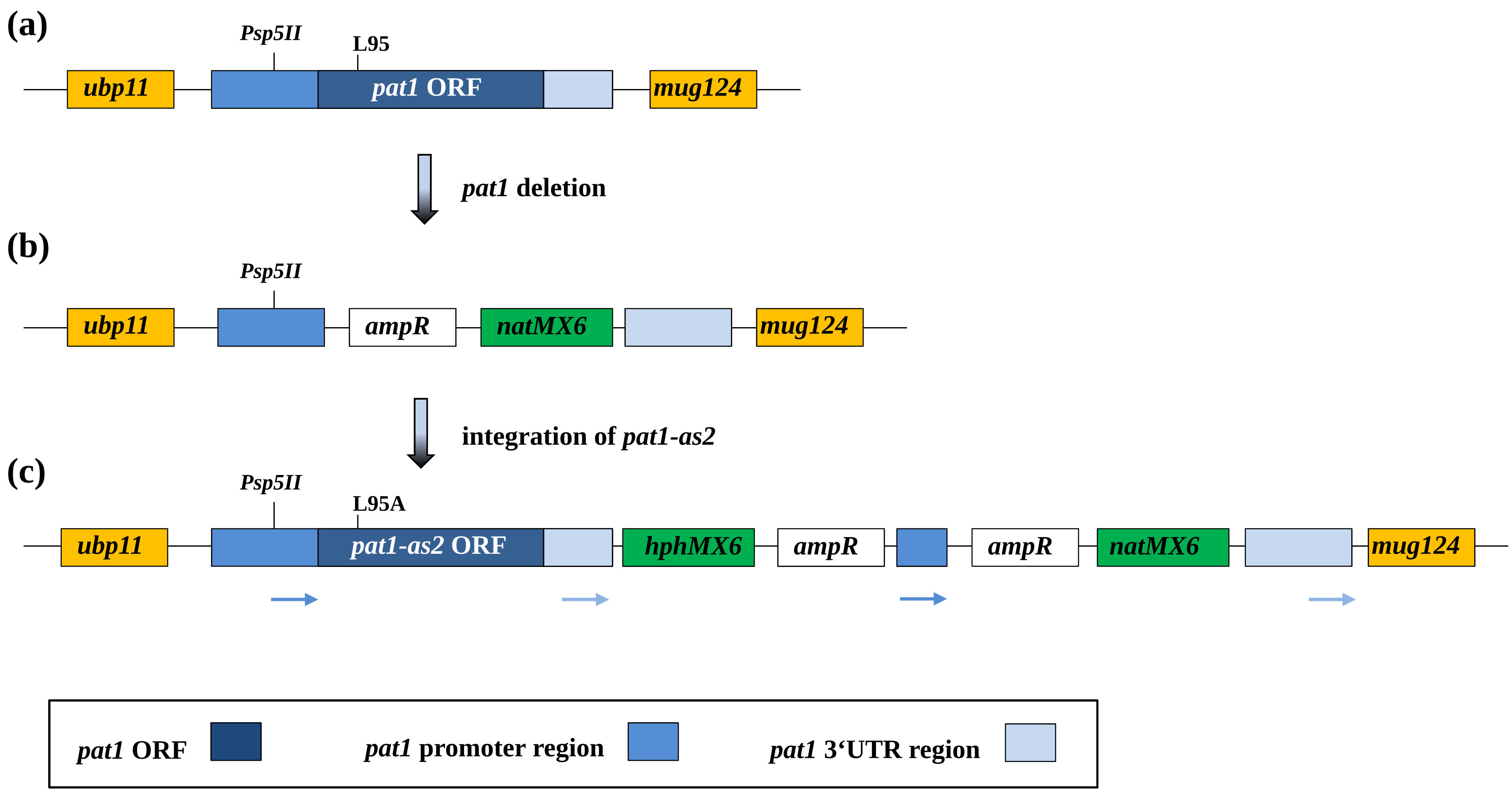
(a)
Psp5II
L95
ubp11
pat1 ORF
mug124
pat1 deletion
(b)
Psp5II
ubp11
ampR
natMX6
mug124
integration of pat1-as2
(c)
Psp5II
L95A
ubp11
pat1-as2 ORF
hphMX6
ampR
ampR
natMX6
mug124
pat1 ORF
pat1 promoter region
pat1 3'UTR region

Supplementary Figure Legends

Supplementary Figure S1. Whole genome DSB distribution in *rad50*$^{+}$ meiosis.
As in Figure 3, DSBs were assayed as Rec12-DNA complexes by immunoprecipitation (IP) of Rec12-FLAG, without artificial cross-linking, amplification of the covalently self-linked DNA, and hybridization to a DNA microarray. IP signals were divided by those from the similarly treated whole cell extract (WCE), median normalized, and plotted for the genome regions indicated; data were further normalized by multiplying the $\log_{10}$ values of the *pat1-as2* signals by 1.1, so that peaks at six DSB sites, shown by Southern blot analysis to be broken to nearly the same extent at the two temperatures (unpublished data), coincided. The DSB hotspot distribution is largely the same in *pat1-as2 rad50*$^{+}$ meiosis at 25°C (red line) and in *pat1-114 rad50*$^{+}$ meiosis at 34°C (black line), apart from the differences highlighted in Figure 3a – h. For 288 DSB hotspots, Pearson's correlation coefficient $r = 0.81$ (Figure S3c).

Supplementary Figure S2. Whole genome DSB distribution in *rad50S* meiosis. DSBs were assayed as in Figures 3 and S1 except that the $\log_{10}$ values of the *pat1-as2* signals at 25°C (red line) were multiplied by 1.1 (for *pat1-as2* at 34°C; blue line) or 1.3 (for *pat1-114* at 34°C; black line) The DSB hotspot distribution is largely the same between *pat1-as2 rad50S* at 25°C (red line) and *pat1-114 rad50S* at 34°C (black line), apart from the differences highlighted in Figure 3a – h; for 288 DSB hotspots, $r = 0.75$ (Figure 3g). The DSB hotspot distribution is indistinguishable between *pat1-as2 rad50S* at 34°C (blue line) and *pat1-114 rad50S* at 34°C (black line); for 288 DSB hotspots, $r = 0.95$ (Figure S3b).

Supplementary Figure 3. Microarray data are highly reproducible. (a) DSB hotspots from both *pat1-as2 rad50*$^{+}$ inductions at 25°C are highly coincident; $r = 0.98$ with the 17 outlying hotspots (Figure 3f) in panel a lying within the range of variation of total hotspots. **(b)** DSB hotspot distributions in *pat1-as2 rad50S* and *pat1-114 rad50S* at 34°C are very similar, indicating that both *pat1* alleles are comparable at high temperature; $r = 0.95$ with the 17 outlying hotspots in panel a lying within the range of variation of total hotspots. **(c)** A second *pat1-as2 rad50*$^{+}$ induction at 25°C shows similar DSB hotspot differences from a *pat1-114 rad50*$^{+}$ induction at 34°C as those in Figures 3a – f. For 288 DSB hotspots, $r = 0.81$, with the

same 11 hotspots having more DSBs at 25°C (blue dots) and six having fewer DSBs at 25°C (red dots), as in Figure 3f. **(d)** The DSB hotspot distributions in *pat1-as2 rad50*$^{+}$ and *pat1-as2 rad50S* at 25°C are very similar; $r = 0.88$ with the 17 outlying hotspots in panel a lying within the range of variation of total hotspots. Data in each panel are plotted as arbitrary units of integrated Rec12-FLAG DSB hotspot signal.

Supplementary Figure 4. Structure of the *pat1* locus in *pat1-as2* strains. (a) Genomic region around the *pat1* gene (wild-type strain). Leucine 95 of Pat1, predicted to be the "gatekeeper" residue is indicated. **(b)** Deletion of the *pat1* gene. DNA flanking the *pat1* gene was PCR-amplified and cloned into a pCloneNat1 vector (EF101285) carrying drug-resistance markers for *E. coli* (*ampR*) and *S. pombe* (*natMX6*). The resulting pCloneNat1-Δ*pat1* plasmid was used to delete one copy of the *pat1* gene in a diploid strain (JG11315) according to the protocol in Gregan et al.). **(c)** Integration of the *pat1-as2* allele. The *pat1-as2* gene together with its promoter and terminator regions was cloned into a pCloneHyg1 vector (EF101286) carrying drug-resistance markers for *E. coli* (*ampR*) and *S. pombe* (*hphMX6*), linearized with Psp5II and integrated next to the *Δpat1* locus of a diploid *Δpat1/pat1*$^{+}$ strain (JG15101) according to the protocol in Gregan et al. (45). Arrows indicate two sections of directly repeated DNA (dark blue and light blue).

References

44. Gregan, J., Rabitsch, P.K., Rumpf, C., Novatchkova, M., Schleiffer, A., and Nasmyth, K. (2006) High-throughput knockout screen in fission yeast. *Nat. Protoc.* 1, 2457-2464.

45. Gregan, J., Zhang, C., Rumpf, C., Cipak, L., Li, Z., Uluocak, P., Nasmyth, K., and Shokat, K.M. (2007) Construction of conditional analog-sensitive kinase alleles in the fission yeast *Schizosaccharomyces pombe*. *Nat. Protoc.* **2**, 2996-3000.
